# Integrin Regulated Autoimmune Disorders: Understanding the Role of Mechanical Force in Autoimmunity

**DOI:** 10.3389/fcell.2022.852878

**Published:** 2022-03-18

**Authors:** Souradeep Banerjee, Ritika Nara, Soham Chakraborty, Debojyoti Chowdhury, Shubhasis Haldar

**Affiliations:** Department of Biological Sciences, Ashoka University, Sonepat, India

**Keywords:** integrin, autoimmune diseases, mechanical force, focal adhesion, tissue stiffness

## Abstract

The pathophysiology of autoimmune disorders is multifactorial, where immune cell migration, adhesion, and lymphocyte activation play crucial roles in its progression. These immune processes are majorly regulated by adhesion molecules at cell–extracellular matrix (ECM) and cell–cell junctions. Integrin, a transmembrane focal adhesion protein, plays an indispensable role in these immune cell mechanisms. Notably, integrin is regulated by mechanical force and exhibit bidirectional force transmission from both the ECM and cytosol, regulating the immune processes. Recently, integrin mechanosensitivity has been reported in different immune cell processes; however, the underlying mechanics of these integrin-mediated mechanical processes in autoimmunity still remains elusive. In this review, we have discussed how integrin-mediated mechanotransduction could be a linchpin factor in the causation and progression of autoimmune disorders. We have provided an insight into how tissue stiffness exhibits a positive correlation with the autoimmune diseases’ prevalence. This provides a plausible connection between mechanical load and autoimmunity. Overall, gaining insight into the role of mechanical force in diverse immune cell processes and their dysregulation during autoimmune disorders will open a new horizon to understand this physiological anomaly.

## Introduction

The concept of “immune tolerance” was proposed by Macfarlane Burnett in 1948, where it was defined as an acquired immunological inertness or “ability of the immune system to prevent itself from targeting self-molecules, cells, or tissues” (Cojocaru et al., 2010). However, further research has discovered that breaches in this tolerance mechanism can lead to the development of autoimmune diseases (ADs), where immune responses against self-antigens are observed. Patients can lead normal lives despite suffering from a single AD with proper lifelong treatment. Additionally, the occurrence of one autoimmune disorder increases the susceptibility for other ADs, which leads to a systematic clinical manifestation called multiple autoimmune syndromes (Cojocaru et al., 2010). This comorbidity brings havoc in the life quality of patients and is predicted to occur in approximately 25% of the population who are suffering from any one AD (Cojocaru et al., 2010). These diseases affect nearly 3%–5% of the population worldwide, and the number is gradually increasing (Jacobson et al., 1997; Eaton et al., 2007; Wang et al., 2015). The onset and prevalence of AD vary among patients as substantial heterogeneity exists by different genetic and environmental factors (Bogdanos et al., 2012; Wang et al., 2015). Nearly a hundred AD have been identified to date, and the list of ADs in the autoimmune registry is being constantly updated (Kienberger et al., 2005). Among them, type 1 diabetes mellitus (T1DM), autoimmune thyroiditis, multiple sclerosis (MS), and rheumatoid arthritis (RA) are some of the most prevalent autoimmune disorders.

It is well-established that mechanical force plays an indispensable role in diverse cellular processes (Webb, 2003; Matsumura, 2005; Vicente-Manzanares et al., 2009; Maître and Heisenberg, 2011; Wruck et al., 2017); however, its direct influence on immune cells and their processes still remains elusive. Different immunological processes, ranging from immune cell migration and adhesion under shear flow to dynamic cell–cell interaction, have been observed to occur under mechanical force (Lafaurie-Janvore et al., 2013; Natkanski et al., 2013; Yusko and Asbury, 2014; Huse, 2017). These forces are sensed as well as transmitted by mechanosensitive proteins present in both the cytosolic and extracellular regions of the cell. Additionally, the nuclear LINC complex and other nuclear proteins such as SUN and YAP/TAZ factors transmit force while interacting with their interactors (Dupont et al., 2011; Bone et al., 2014; Elosegui-Artola et al., 2017; Donnaloja et al., 2019). Mechanosensitive ion channels such as different subtypes of transient receptor potential (TRP) channel (Nikolova-Krstevski et al., 2017), the mechanosensitive channel of small conductance (MscS) channels (Zhang et al., 2021), and piezo channels (Wang et al., 2019) have been reported to be involved in MS and experimental autoimmune encephalomyelitis (EAE) model, RA (Jairaman et al., 2021), ulcerative colitis (Toledo-Mauriño et al., 2018; Silverman et al., 2020), and Crohn’s disease (Alaimo and Rubert, 2019). These mechanosensitive proteins sense force and subsequently transduce biochemical signals to both inside and outside of the cell, regulating cell shape, size, and its fate (Dong et al., 2009; Paluch and Heisenberg, 2009; Yusko and Asbury, 2014; Sivarapatna et al., 2015; Kumar et al., 2017; Leal-Egaña et al., 2017; Schakenraad et al., 2020). Among these mechanosensitive proteins, adhesive proteins are the major players in mediating the mechanical cross-talk between the cell and extracellular matrix (ECM). Integrin, being a major adhesive protein, plays a crucial role in AD progression through different immune cell processes (McMurray, 1996; Steinman, 2004; Rose et al., 2007; Chase et al., 2012; Engl et al., 2014; Sun et al., 2016). While interacting with both the intracellular and intercellular partners, integrin regulates immune cell functioning like cell migration, adhesion, lymphocyte activation as a major co-stimulator (Kong et al., 2009; Sun et al., 2016; Nordenfelt et al., 2017; Jaumouillé et al., 2019). Notably, force plays a regulatory role in integrin activation, and several studies have quantified the mechanical force controlling the integrin-mediated immune mechanisms (Woolf et al., 2007; Kong et al., 2009; Chen et al., 2010; Franck et al., 2011; Stout et al., 2016; Sun et al., 2016; Sun et al., 2019). Therefore, the mechanical role of integrin in the causation of abnormal immune responses, specifically in AD, is of keen interest. In this review, we have illustrated how integrin’s mechanosensitivity is regulated in different immune cell processes, resulting in different ADs. Interestingly, we have provided a new insight that tissue stiffness possesses a positive correlation with AD prevalence, indicating a plausible role of tissue stiffness in AD progression. Overall, this review will provide a new physical perspective to autoimmune disorders, where mechanical load could play a pivotal role in disease pathobiology.

### INTEGRIN SENSING MECHANICAL FORCE

Ligand specificity of integrins is decided by the couplet combinations of its α and β subunits (Table 1). Generally, one integrin heterodimer is capable of binding many ligands, and similarly, one ligand can interact with different integrin subtypes. Extracellular ligand interactions of integrin are divided into several groups, based on the structural disposition of the molecular interaction (Hynes, 2002; Humphries et al., 2006; Bachmann et al., 2019): i) RGD-binding integrins, recognizing diverse extracellular ligands with RGD motif; ii) LDV motif-binding integrins, which interact with ligands with LDV motif; iii) αI domain-containing α subunits, which bind to laminin/collagen; iv) non-αA/αI domain-containing integrin, which interacts with laminin while pairing with β1 subunit (Humphries et al., 2006); and v) some integrins that exhibit a change in conserved GFFKR sequence in the membrane proximal part of α subunit (Dickeson and Santoro, 1998; Hynes, 2002; Humphries et al., 2006; Barczyk et al., 2009; Bachmann et al., 2019). On the other hand, members of the integrin interactome can be broadly classified into three categories: ECM ligands containing the RGD sequence; transmembrane proteins such as tetraspanin, syndecan, and CD47, which interact laterally with integrins while being attached to the cell membrane; and intracellular proteins like talin and kindlin binding to the cytosolic tails of α and β subunits to trigger inside-out signaling (Emsley et al., 2000; Xiong et al., 2002; Shimaoka et al., 2003; Xiao et al., 2004).

**TABLE 1:** Classification of major integrin with a cluster of differentiation (CD) nomenclature.

β subunit	α subunit	Integrin name	Classification based on binding site	Classification based on structure	Major ligands	Expression
β1 (CD29)	α1 (CD49a)	α1β1 (VLA-1)	LDV binding	αI domain containing	Laminin, collagen, tenascin	NK cells activated B and T cells
	α2 (CD49b)	α2β1 (VLA-2)		αI domain containing	Laminin, collagen	NK cells activated B and T cells
	α3 (CD49c)	α3β1 (VLA-3)		XGFFKR sequence containing	Laminin, collagen, fibronectin	Thymocytes and activated T cells
	α4 (CD49d)	α4β1 (VLA-4)			Fibronectin, VCAM1, MAdCAM1, TSP-1	Monocytes and lymphocytes
	α5 (CD49e)	α5β1 (VLA-5)	RGD specific		Fibronectin, L1	Macrophages
	α6 (CD49f)	α6β1		XGFFKR sequence containing	Laminin	T cells (memory and activated), thymocytes
	αv (CD51)	αvβ1	RGD specific		Vitronectin, fibronectin, collagen, fibrinogen	T regulatory cells
β2 (CD18)	αL (CD11a)	αLβ2 (LFA-1)		αI domain containing	ICAM1, 2 and 3	All leukocytes and is predominant in lymphocytes
	αM (CD11b)	αMβ2 (Mac-1)		αI domain containing	ICAM1, iC3b, fibrinogen	Especially neutrophils and monocytes also expressed in NK cells, B cells, and some T cells
	αX (CD11c)	αXβ2		αI domain containing	iC3b and fibrinogen	Myeloid dendritic cells (DCs)
	αD (CD11d)	αDβ2		αI domain containing	ICAM-3, VCAM1	Eosinophils, neutrophils, monocytes, and NK cells
β3 (CD61)	αv (CD51)	αvβ3	RGD specific		Fibronectin, osteopontin, PE-CAM1, vitronectin, fibrinogen, human L1, thrombospondin, collagen	Monocytes activated B and T cells
	αIIb (CD41)	αIIbβ3	RGD specific		Fibronectin, vitronectin, thrombospondin	Mast cells
β5	αv (CD51)	αvβ5	RGD specific		Vitronectin, fibronectin, fibrinogen	Monocytes and macrophages
β7	αE	αEβ7 (CD103)		αI domain containing, XGFFKR sequence containing	E-cadherin	Mainly expressed on mucosal T cell
	α4 (CD49d)	α4β7	LDV binding		Fibronectin, VCAM1, MAdCAM-1	Circulating lymphocytes

Note. Classification of the integrin subtypes with structural features and/or their binding sites on respective ligand molecules. Classification is based on data from (Dickeson and Santoro, 1998; Humphries et al., 2006; Barczyk et al., 2009; Bachmann et al., 2019). CD nomenclatures are according to the Human Cell Differentiation Molecules (https://www.hcdm.org/).

CD, cluster of differentiation; LDV, a motif of some integrin ligands; RGD, a motif of the majority of integrin ligand; αI domain, a chordate specific domain in the α subunit of integrin; XGFFKR, a sequence present in the proximal cytoplasmic tail of integrin α subunit where X is a variable amino acid.

### Integrin-Talin Centered Focal Adhesion

**FIGURE 1:**
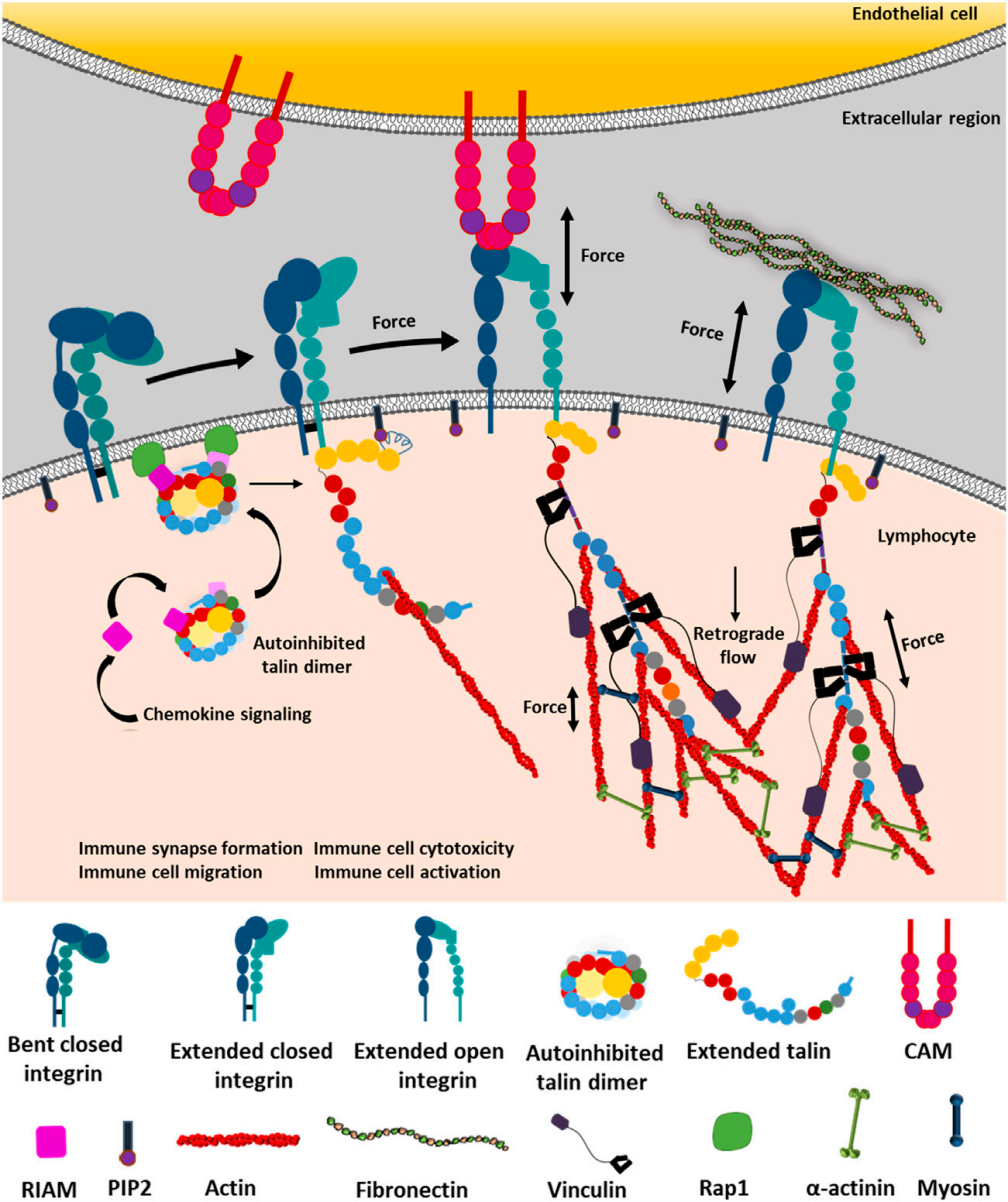
Integrin-dependent mechanotransduction by outside-in and inside-out signaling. Integrin can exist between three conformational states: bent-closed, extended-closed, and extended-open conformation. Bent-closed conformation is functionally inactive and thus could not interact with cell–extracellular matrix (ECM) ligands. Chemokine signaling initiates RIAM to bind the autoinhibited talin. The autoinhibited talin–RIAM complex binds to the Rap1 protein, which activates talin by extending it from the autoinhibited structure. Subsequently, the extended talin binds to the NPxY motif of the cytosolic tail of the β subunit of integrin. Talin binds to PIP2 by the FERM domain (red pentagon) and actin by its actin-binding domains. These interactions break the transmembrane salt bridge between α and β subunits and activate integrin by providing the required force, which allows integrin to cross its internal thermodynamic barrier, resulting in the active state stabilization by the very low force provided by talin. Now activated integrin is able to bind ECM ligands on the extracellular region connected to the actomyosin complex inside the cell. On the contrary, integrin also gets activated from the extended-closed structure through outside-in force sensing by forming interacting bonds with its intercellular ligands like CAMs or ECM proteins. The thermodynamic barrier causes conformational fluctuation between the most stable bent-closed to unstable extended-open conformation through a transient extended-closed state. While experiencing ligands outside the cell, the extended-closed conformation has the ability to form a transient bond with the ligand (here CAM), which transmits the force through integrin to talin. Talin along with PIP2 breaks the transmembrane salt bridge, activating the integrin to extended-open conformation. This is followed by the binding of the actin cytoskeleton to talin. This provides longer and more durable catch-bond formation, under force, between the integrin-extracellular ligand, thus transducing the signaling cascades and retrograde flow to regulate immune synapse formation, activation of lymphocytes, tissue invasion by migration, cytotoxicity, etc. (Orłowski et al., 2015b; Haining et al., 2016; Yao et al., 2016; Khan and Goult, 2019; Sun et al., 2019).

**FIGURE 2:**
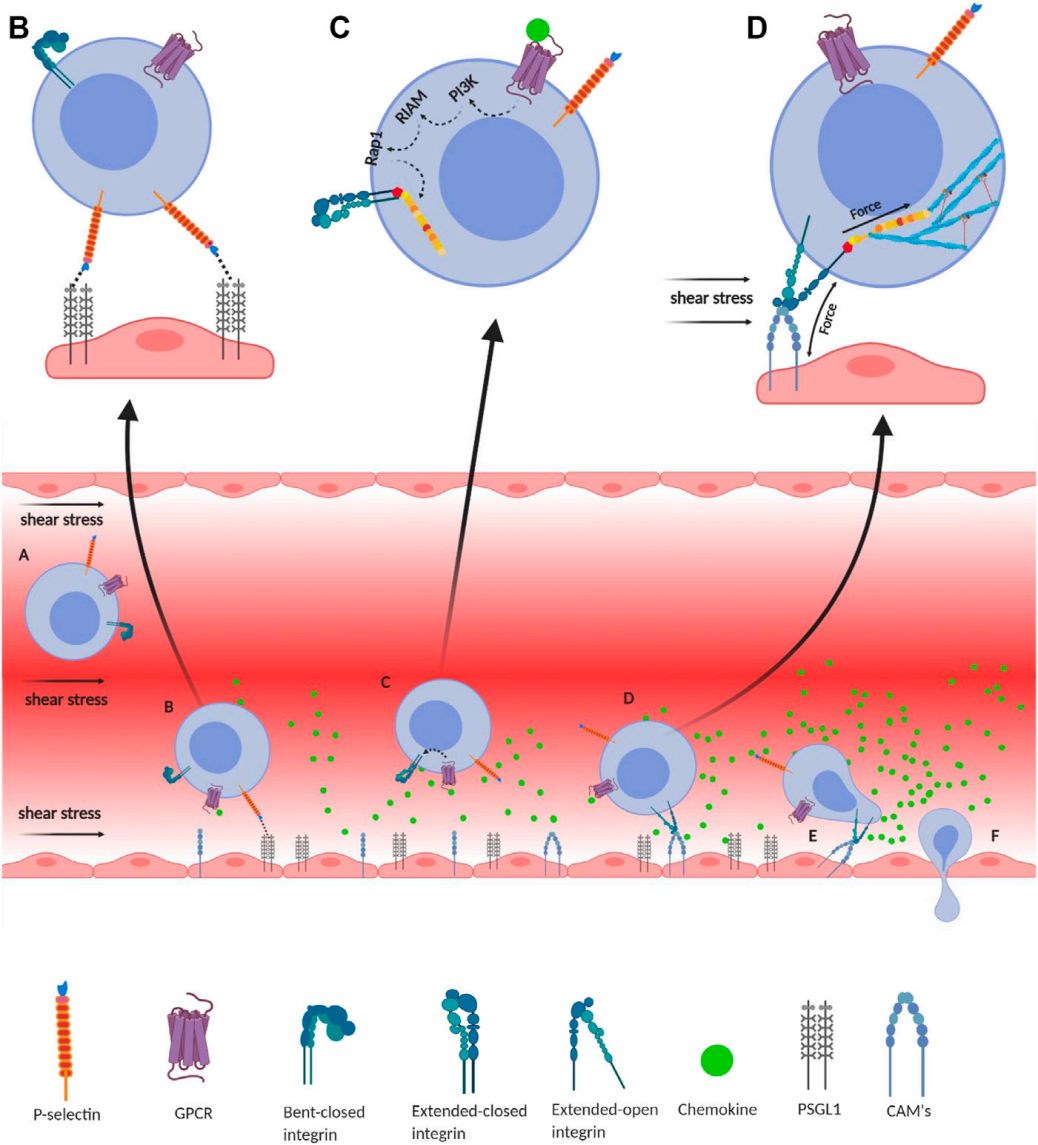
Integrin-mediated immune cell adhesion to endothelial cells under a shear force of blood flow. **(A)** Migration of immune cells under force—immune cells traveling through the blood vessel experience a shear force of the blood flow. Chemokines (green) are secreted by the endothelial cells lining the tissue displaying self-antigens; however, the chemokine gradient is highest near the infectious tissue. The chemokines slow down the flow rate of the migratory leukocytes towards the site of infection under the shear stress of blood flow, equivalent to 1 dyn/cm^2^. **(B)** Slip-bond formation and decrement in cell migration velocity—cells gradually decrease the speed along with the rise of chemokine gradient and tumble on the endothelial cells of the blood vessel. The selectin molecules, expressed by the leukocyte, interact with its counterpart expressed on the endothelial cells. However, their interaction under a shear force of blood flow causes the slippage of the bonds, allowing the cell to roll on the endothelial layer, while rolling numerous numbers of slip bond forms and breaks between the molecules like P-selectin, E-selectin, PSGL1, E-cadherin, etc. **(C)** Extended-closed integrins—the GPCR expressed on the leukocytes interacts with the chemokine to activate PI3K that induces Rap1–RIAM complex to activate talin for further binding with the β subunit cytosolic tail of integrin. This partially activates integrin from its bent-closed to extended-closed structure. **(D)** Integrin activation leading to focal adhesion—the extended-closed integrin gets activated, either by outside-in signaling by interacting with CAM while rolling on the endothelial layer or by inside-out signaling through sensing the force from talin–actin complex. The activation breaks the integrin salt bridge, transforming it into a thermodynamically unstable but active extended-open conformation. This forms integrin–ligand catch bonds under blood-flow shear force, resulting in complete adhesion of the immune cells to the endothelial layer. During this interaction, the force is transmitted through integrin both outside and inside the cell, which finally transduces downstream forming the focal adhesion. **(E)** Adhesion of cell—this focal adhesion regulates the cell's shape and migration and strictly adheres the cell on the endothelial layer by inducing the catch-bond formation. **(F)** Diapedesis—while remaining attached on the endothelial surface in the infected tissue, the self-reactive immune cells transmigrate in between adjacent cells by diapedesis towards the infected tissue region (Zhu et al., 2007b; Jahed et al., 2014; Huse, 2017).

Integrin subtypes undergo conformational changes through three states: bent-closed, extended-closed, and extended-open conformation. However, its underlying mechanism upon ligand binding is highly debated by the supporters of switchblade and deadbolt models. Integrin activation, shifting from its bent-closed conformation (inactive) to the extended-open conformation (active with high affinity), causes the ligand-binding site to move 150–200 Å away from the cell surface (Zhu et al., 2007a; Jahed et al., 2014). This is followed by the initiation of integrin-mediated mechanotransduction by switching to its thermodynamically unstable active conformation by either “outside-in” or “inside-out” mechanism. The “inside-out” mechanism involves a key intracellular player talin, which, along with kindlin, has the unique ability to activate integrins (Goult et al., 2021; Cowell et al., 2021). This activation of integrins, followed by ligand binding, results in integrin clustering. This causes the heterodimers to oligomerize, forming lateral assemblies that eventually mature into focal adhesion complexes (Jahed et al., 2014). Though the mechanism of clustering is elusive, it is majorly regulated by inside-out signals that recruit multimeric protein complexes to integrin tails (Shattil et al., 2010). By contrast, outside-in signaling allows integrin to bind ECM proteins such as fibronectin, laminin, and collagen, enhancing the force transmission across the cell membrane and subsequent integrin interaction with talin and kindlin (Sun et al., 2019; Chakraborty et al., 2019; Goult et al., 2018). Once talin binds to the NPxY motif in the structurally conserved PTB-like domain of integrin, integrin α and β cytoplasmic tails separate, resulting in its activation (Kong et al., 2009). Interestingly, it is recently discovered that the flexible loop in the F1 domain of the integrin head is crucial for activating the β3 domain of integrin (Kukkurainen et al., 2020). Although talin itself is unable to cross the thermodynamic barrier to activate integrin, it can disrupt the transmembrane salt bridge between two integrin subunits with the help of PIP_2_ (Figure 1) (Sun et al., 2019; Orłowski et al., 2015a). Talin remains attached to the cytoskeleton via actin and acts as a linchpin partner for integrin in relaying force from the inside-out (Cowell et al., 2021; Chakraborty et al., 2019). Talin interacts with the RIAM protein in a Rap1-dependent manner and has been observed to enhance integrin activation during leukocyte stimulation (Goult et al., 2013; Yao et al., 2015; Gough et al., 2021; Han et al., 2006). Kindlin, on the other hand, binds to the membrane-distal region of the β-integrin tail to its NxxY motif. While a tension of 10 pN has been measured across talin molecules at focal adhesion sites, kindlin experiences no intramolecular tension despite being directly linked to F-actin (Austen et al., 2015; Bledzka et al., 2016). Both protrusive and contractile F-actin dynamics work in tandem at cell–ECM contacts to generate frictional drag (Huse, 2017). These molecules form the focal adhesion complex with talin–integrin linkage as a center of the “molecular clutch.” Gradual integrin clustering matures the focal adhesion by recruiting adaptor proteins like vinculin and kindlin, manipulating actin retrograde motion by traction force generation (Figures 1, 2D,E) (Khan and Goult, 2019). Remarkably, increased forces sustained by the focal adhesion have been shown to correlate with the integrin cluster size during the focal adhesion maturation for larger adhesions over 1 μm (Wang et al., 2015). No such correlation, however, exists for smaller adhesions or beyond the initial stages of myosin-mediated adhesion maturation and growth (Tan et al., 2003; Stricker et al., 2011; Mehrbod and Mofrad, 2013).

Each talin–integrin molecular clutch is believed to have its own threshold, beyond which a mechanosensing event is triggered resulting in the adhesion growth by increased integrin recruitment (Oria et al., 2017). The entire dynamics are tightly controlled by mechanical signals, acting as a well-oiled “gearbox.” As a result, the adhesion turnover is monitored through the contraction of the actomyosin skeleton and the cellular traction force (Chakraborty et al., 2019). The rate of adhesion turnover is essential in the force transmission and adhesion strengthening, since it controls the force redistribution pattern across its scaffolding thereby, forming a heterogeneous focal adhesion complex (Elosegui-Artola et al., 2016). Interestingly, the cellular response increases with both matrix rigidity and ligand density, which finally promotes adhesion growth (Elosegui-Artola et al., 2016). This challenges the wide consensus where the collapse of the adhesion complex was observed under high load, beyond a second rigidity threshold of 30 kPa for 100-nm-spaced substrates and 150 kPa for 50-nm-spaced substrates (Oria et al., 2017). Additionally, a small increase in ECM stiffness can directly affect mechanotransduction (DuFort et al., 2011). For example, on soft ECMs (∼1.5 kPa), integrins cluster with intermolecular distances of ∼200 nm (Oria et al., 2017), but stiffer ECMs of higher tensions (∼150 kPa) enable denser clustering of integrins with ∼60-nm separable distance forming more stable adhesions (Cavalcanti-Adam et al., 2007). Interestingly, the positioning of molecular clutch engagement varies among cell types and affects those cellular motilities (Huse, 2017). Hence, the talin–integrin clutch plays a crucial role in efficient migration by localizing the adhesions to areas with stiff ECM and active F-actin protrusion. This additionally constrains the rapid actin polymerization, which otherwise is energetically costly and limits the formation of unnecessary adhesive contacts (Huse, 2017).

The role of force-dependent integrin binding in cell–cell adhesion and cell–ECM interaction is indispensable. Different force-based imaging techniques have observed the biomechanics of leukocyte circulation, endothelial and *trans*-endothelial migration, and their persistence in the surrounding matrix (Schwartz et al., 2021). For example, traction force microscopy (TFM) has revealed that neutrophils and migrating T cells have force exertion concentrated in the rear side, where fully activated extended integrins are also found to cluster, similar to a “rear-wheel drive” mechanism (Jannat et al., 2011a; Dixit et al., 2011; Smith et al., 2007; Green et al., 2006). By contrast, macrophages and dendritic cells (DCs) exhibit maximum traction forces near the leading edge of the diapadesing cell (Gardel et al., 2010a; Renkawitz and Sixt, 2010a). This is similar to the “front-wheel drive” of fibroblasts and endothelial cells (ECs), which form focal adhesions at the base of their lamellipodia (Gardel et al., 2010b; Renkawitz and Sixt, 2010b). Leukocyte diapedesis has been shown to increase with the substrate stiffness, which in turn is correlated with higher DLC-1 expression in ECs. This stabilizes ICAM1 (a ligand of LFA-1 and Mac-1) adhesome during the *trans*-endothelial migration, a form of diapedesis (Schimmel et al., 2018). It is also well known that *trans*-endothelial migration of leukocytes is strongly enhanced by the matrix stiffness of the vasculature (Huynh et al., 2011). Notably, α actinin-4 recruitment has been reported to be a strong influencer of endothelial stiffness, regulating the spreading and subsequent diapedesis efficiency of adhesive polymorphonuclear (PMN) cells. This EC stiffness also regulates the function of ICAM1, an integrin ligand, controlling the transmigration of neutrophils (Schaefer et al., 2014). Martinelli et al. has shown that EC mechanics including a defined substrate stiffness can switch the diapedesis route (Martinelli et al., 2014). Indeed, they observed that initiation of diapedesis requires local reduction of EC stiffness, and thus, *trans*-endothelial migration occurs majorly at low stiffness sites (Huveneers et al., 2015). Recently, it has been shown that monocyte migration and adhesion are also stiffness dependent and correlate well with ICAM1/VCAM1 expression (Chen et al., 2019). The mechanism by which ECs render the matrix stiffness toward *trans*-endothelial migration remains less explored. An AFM-based study shows that increasing the matrix stiffness from 0.5 to 100 kPa increases LFA-1/ICAM1 binding force from 123 to 220 pN, thereby augmenting the chance of leukocyte adhesion to ECs and promoting *trans*-endothelial migration (Jiang et al., 2016). Monocyte adhesion and diapedesis have been shown to be dependent on integrin ligands such as ICAM1, ICAM2, and VCAM1 (Schenkel et al., 2004). Neutrophil transmigration has also been reported to be influenced by EC stiffness through myosin light chain kinase (MLCK)-dependent cell contraction (Stroka and Aranda-Espinoza, 2011). By contrast, in the case of ICAM1 or VCAM1 interaction, CD4^+^ T-cell migration becomes shear dependent instead of stiffness dependent (Kim and Hammer, 2021). Similarly, another study has shown a stiffness-dependent T-cell migration and adhesion via T-cell receptor (TCR) mechanosensing (Bashour et al., 2014). Inflammation can result in higher stiffness of the tissue matrix, further modulating the transmigration pathway (Fowell and Kim, 2021). This indicates that the endothelial stiffness effect on transmigration could be a linchpin factor depending on the cell type interacting with the ECs with respective ligand interactions. Additionally, tenertaxis, or the guidance of lymphocyte migration by the path of least mechanical resistance, has been proved to support the lymphocyte diapedesis through the mechanically softer tissues (Martinelli et al., 2014). Interestingly, leukocyte migration through 2D and 3D environments differs according to the matrix and tissue stiffness (Mestas and Ley, 2008; McIntyre et al., 2003). For example, leukocytes, although displaying adhesive receptor-dependent migrations in 2D, generally prefer amoeboid-type migration in 3D, which is independent of adhesion proteins (Gaertner et al., 2022; Yamada and Sixt, 2019; Reversat et al., 2020). However, mesenchymal migration of macrophages has been reported to be adhesion protein-dependent with integrin as a major one. Cui et al. (2018) showed that macrophage migration can be regulated by αMβ2 and αDβ2 integrin-mediated adhesome even in a 3D environment, and thus, receptor-mediated migration is not only limited to 2D matrix stiffening. Recently, Bhattacharjee et al. discussed that immune cell–ECM crosstalk could be critically involved in different autoimmune skin diseases (Bhattacharjee et al., 2019). Different groups have debated that immune cell–ECM interaction is pivotal for cell migration and other immune cell processes (Boyd and Thomas, 2017; McMahon et al., 2021; Moreau et al., 2017). Hons et al. (2018) has also demonstrated that intra-nodal migration of T cells is regulated by both cytokine and integrin, controlling actin flow and substrate friction. Other ADs (except RA) like scleroderma and psoriasis are known to be crucially regulated by integrin interaction with matrix ligands (Conrad et al., 2007; Pattanaik et al., 2015; Gerber et al., 2013). As the mesenchymal migratory route is opted more often in the stiffened matrix, with the help of matrix metalloproteinase (MMP)-secreting invadopodia, the stiffened matrix also regulates the occurrence of pathobiological signaling. Specialized cellular structures like podosomes and invadosomes, which are involved in diapedesis, invasion, and migration of myeloid-originated immune cells (Dufrançais et al., 2021), are formed by the integrin-mediated focal adhesion complex (Martinelli et al., 2014; Hood and Cheresh, 2002). Labernadie et al. measured the podosome mechanics within the living macrophage using AFM methodology and observed that the podosome stiffness is 43.8 ± 9.5 kPa (reported as mean ± s.e.m.). This specialized cellular structure is crucial in assisting the motility of macrophages through ECM degradation and tissue invasion (Labernadie et al., 2010). Integrin-controlled immune cell processes mentioned here and in Table 2 support the role of mechanotransducing integrin in inflammatory processes, which finally assists the immune cells in migration and tissue penetration (Hood and Cheresh, 2002).

**TABLE 2:** Integrin–ligand interaction playing regulatory roles in immune cells processes.

Immune processes	Integrin types	Integrin–ligand interactions	Force quantified in these interactions	References
Lymphocyte migration	α4β1 (VLA-4)	α4β1/VCAM1	∼50 pN	Chan and Aruffo (1993), Zhang et al. (2004)
			AFM-based study [10 pN/s (Wang et al., 2015) to 10 pN/s (Bogdanos et al., 2012)]	
	αLβ2 (LFA-1)	αLβ2/ICAM1	10–15 pN	Chen et al. (2010), Piechocka et al. (2021)
			Biomembrane force probe-based study	
Eosinophil adhesion	αDβ2	αDβ2/VCAM1	NA	Grayson et al. (1998)
Monocyte migration	αDβ2	NA	NA	Yakubenko et al. (2008)
Lymphocyte homing	α4β7	α4β7/ MAdCAM1	32–80 pN	Sun et al. (2014), Wang et al. (2018)
			AFM-based study (100–1,500 pN/s)	
Macrophage differentiation	α5β1	α5β1/fibronectin	10–30 pN	Laouar et al. (1999), Kong et al. (2009)
			AFM-based study	
T-lymphocyte adhesion	αLβ2 (LFA-1)	αLβ2/ICAM1	10–15 pN	Sigal et al. (2000), Chen et al. (2010)
			Biomembrane force probe-based study	
	αEβ7	αEβ7/E-Cadherin	60 pN	Taraszka et al. (2000), Shibata-Seki et al. (2020)
			AFM-based study	
Macrophage adhesion	α5β1	α5β1/Fibronectin	10–30 pN	Kong et al. (2009), Evans et al. (2019)
			AFM-based study	
	αDβ2	αDβ2/vitronectin	NA	Yakubenko et al. (2008)
Formation of immunological synapse (IS) or supramolecular activation cluster (SMAC) in T cell	αLβ2 (LFA-1)	αLβ2/ICAM	10–15 pN of biomembrane force probe-based study	Monks et al. (1998), Chen et al. (2010)
B-cell adhesion, activation, and synapse formation	αLβ2 (LFA-1)	αLβ2/ICAM1	10–15 pN	Carrasco et al. (2004), Chen et al. (2010)
			Biomembrane force probe study	
Neutrophil crawling	αMβ2 (Mac-1)	αMβ2/ICAM1	10 pN	Phillipson et al. (2006), Rosetti et al. (2015)
			Biomembrane force probe-based study	
Monocyte and platelet adhesion	αMβ2	αMβ2/CD147	NA	Heinzmann et al. (2020)
Inflammatory response	αMβ2 α4β1	αMβ2/pleiotrophin	NA	Feng et al. (2021)
AFM-based study (10 pN/s (Wang et al., 2015) to 10 pN/s (Bogdanos et al., 2012)	(VLA-4)	α4β1/VCAM1	∼50 pN	Zhang et al. (2004), Lou et al. (2021)
Complement activation	αMβ2	αMβ2/iC3b	NA	Xu et al. (2017)
	αXβ2	αXβ2/iC3b	NA	Xu et al. (2017)

### Mechanical Interactions Between Integrin and Their Respective Ligands

It is well-established that integrins sense and transmit mechanical force; however, it remains unclear whether a specific integrin bears maximum load (over 30 pN) or it is a cumulative effort of many weaker interactions by the entire adhesion structure (Chang et al., 2016). As integrin activation and ligand binding result in integrin clustering on the cell membrane, hundreds of adaptors and signaling molecules nucleate at their cytosolic tails to form a large dynamic supramolecular complex, called the integrin adhesome or focal adhesion (DuFort et al., 2011). Single-molecule techniques like FRET-based molecular tension sensor (Li and Springer, 2017), AFM (Hinterdorfer et al., 1996), optical force microscopy (Stout and Webb, 1998), magnetic tweezers (Roca-Cusachs et al., 2009), and ensemble techniques like micropipette-based force transducers (Evans et al., 1991; Evans et al., 1995; Shao and Hochmuth, 1996; Chesla et al., 1998), centrifugation (Lotz et al., 1989; Piper et al., 1998), and shear flow have been used to measure the integrin–ligand interaction under force (Tha et al., 1986; Alon et al., 1995; Pierres et al., 1995a; Pierres et al., 1995b). An AFM study by Franz et al. has observed receptor–ligand recognition forces to fall within the wide range of 1–100 pN at a loading rate of 10^2^–10 pN/s (Bogdanos et al., 2012), and acting on short distances between 0.1 and 1 nm (Franz et al., 20072007). Recently, Chang et al. (2016) observed that most integrins bear 1–7 pN of force, which is nearly 10-fold less than the maximum load that integrins have been found to uphold. By contrast, a previous AFM study showed that a peak rupture force of 120 pN (observed at a loading rate of 10–50,000 pN/s and pulling speed of 1–15 μm/s) is required for a single α5β1/FN interaction (Li et al., 2003). However, using optical tweezers, Thoumine et al. (2000) measured average integrin bond strength within 20–28 pN. Interestingly, it has also been observed that some integrin subtypes within the adhesions have the ability to withstand higher forces than the empirical measurement, reinforcing the idea of differential force transmission among integrin subtypes. In fact, when fibronectin-binding α5β1 and αVβ3 were subjected to a small force of 1 nN using magnetic tweezers, Roca-Cusachs et al. (2009) found that αVβ3 could not sustain the applied forces while α5β1 was inhibited, suggesting individual integrin molecules are capable of withstanding different mechanical loads. While αVβ3 is important for reinforcement and mechanotransduction, α5β1 is mainly involved in mediating adhesion strength (Roca-Cusachs et al., 2009). At 30 pN, α5β1 integrin achieves maximal affinity for fibronectin (Kong et al., 2009), while LFA-1 and Mac-1 show optimal functioning under 10–15 pN (Chen et al., 2010; Rosetti et al., 2015). Moreover, using AFM-based single-cell force spectroscopy, Wang et al. (2018) suggested ligand-specific activation of α4β7 *via* MAdCAM-1 and VCAM1 interactions and showed that Mn^2+^ addition increased the force-dependent lifetime of these interactions besides increasing integrin ligand-binding affinity. The ability of α4β7 to switch its conformer specificity allows it to precisely regulate leukocyte homing in tissue. These data also suggested that β2 integrin may also have similar ligand-specific active states induced by differential activation (Wang et al., 2018). Therefore, the force spectroscopic technologies quantified the force-dependent interactions of different integrins with their ligands, which further aided in understanding their interactions *in vivo.*

In addition to biochemical and intracellular activation, integrins can also be activated by forces experienced directly from the extracellular region, inducing catch-bond formation with the respective ligand. While most tensional forces weaken protein–protein interactions by forming slip bonds (Figure 2B), catch bonds are formed between almost every integrin–ligand interaction. By definition, catch bonds are formed between receptor and ligand to act like molecular hooks that dissociate easily in the absence of force but remain reinforced under tensile forces (Hertig and Vogel, 2012). These bonds are induced upon experiencing a range of mechanical force and are responsible for strengthening adhesion and drastically increasing bond lifetimes. For example, for specific interaction between α5β1 and fibronectin, 10–30 pN of force was observed by Kong et al. (Sun et al., 2019). However, while force application accelerates catch-bond activation by passing the short- to long-lived state of integrin across its free energy barrier, it is not essential for strengthening adhesion (Hertig and Vogel, 2012; Kinoshita et al., 2010; MacKay and Khadra, 2020). In the case of integrin, many extracellular domains can interact with each other when in bent-closed conformation to stabilize the nonactivated state (Hertig and Vogel, 2012). More importantly, the catch bond formed between α5β1 and fibronectin leads to a force-induced conformational change in the integrin headpiece allosterically, which drives the α5 subunit to associate with the synergy site in FNIII9 of fibronectin (Figure 2D) (Kong et al., 2009; Chen et al., 2010; Rosetti et al., 2015; Sun et al., 2019). Kong et al. (2009), using AFM-clamp experiments, quantified the lifetime of single α5β1/FN bonds at forces as low as 4 pN and observed catch-bond formation ≤30 pN (at a cantilever pulling speed of 200 nm/s). Upon truncating the leg region and using two activating monoclonal antibodies (mAbs) binding the headpiece, they found that the catch-bond formation involves force-assisted activation of the headpiece but not integrin extension (Kong et al., 2009). Additionally, integrins like LFA-1 (αLβ2) and Mac-1 (αMβ2) also form catch bonds with their ICAM ligands. Notably, Lou et al., using a biomembrane force probe, measured single-bond interactions between LFA-1 and ICAM1 (Chen et al., 2010). They found that integrin LFA-1 forms catch–slip bonds with ICAM1 in three cation conditions and in the presence of a chemokine that triggers inside-out signaling. With a gradual increment of force, LFA-1/ICAM1 bond lifetimes first increase, forming catch bonds, and as their off-rates decrease, then slip bonds form beyond a threshold of 15 pN, declining the bond lifetime (Chen et al., 2010). Interestingly, on changing the divalent cations from Ca^2+^/Mg^2+^ to Mn^2+^, the peak of the average lifetime curve has been observed to increase from 10 to 15 pN. More importantly, upon using an internal ligand antagonist XVA143 that blocks the pulling force of the α7-helix, suppression of intermediate-/long-lived states was observed, leading to the elimination of catch bonds and revealing an internal catch bond between the αI and βI domains of LFA-1 (Chen et al., 2010). In contrast, to catch bonds, a more intuitive biomolecular interaction is the formation of slip bonds. These slip-bonds can be observed between E-selectin and their ligands, like different integrins, antibodies or antigens. (Li et al., 2016). Chen et al. (2011) showed that pulling force at a cyclic RGD motif bound to the integrin head also extended the integrin, suggesting force-dependent activation of integrins. The formation of catch bonds between integrins and their ligands is proved to be an important aspect of various immunological functions. For example, the LFA-1/ICAM1 interaction is majorly responsible for leukocyte migration and firm adhesion under force (Chen et al., 2010). Similarly, the fibronectin-receptor integrin α5β1 plays a direct role in angiogenesis (Kong et al., 2009). Integrin α4β1 (or VLA-4) is expressed on T and B lymphocytes, monocytes, eosinophils, neutrophils, and natural killer cells, promoting inflammatory responses by assisting leukocyte migration. Lastly, αMβ2 (or Mac-1) is another important integrin that is highly upregulated in migrating phagocytes (Lishko et al., 2003). These examples lead to the understanding that catch bond–slip bond transitions during the integrin–ligand interactions, under mechanical force-sensitive scenarios, will play crucial roles in immune cell mechanisms.

## Mechanosensitive Integrins Regulate Immune Cell Processes

**FIGURE 3:**
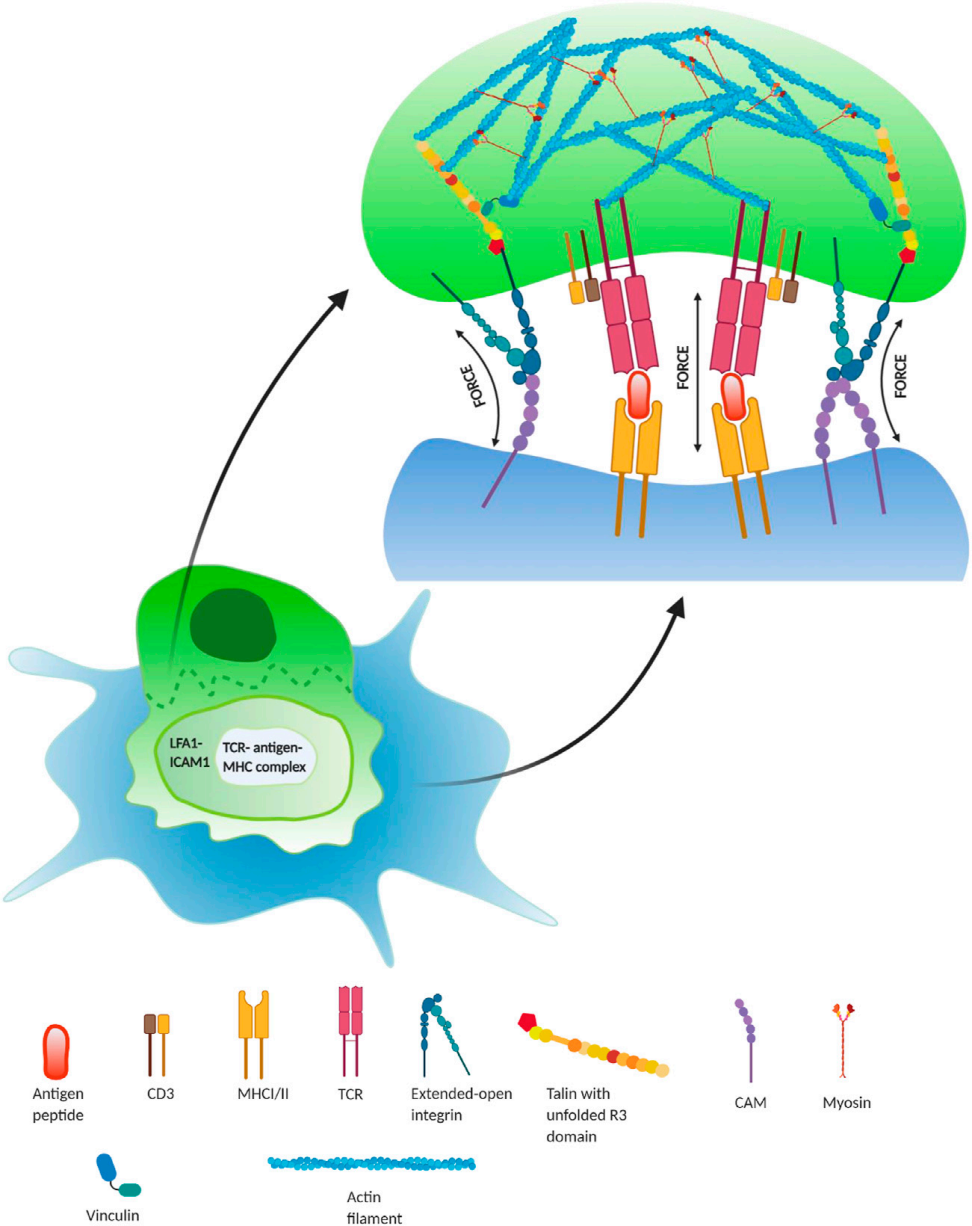
Regulatory role of force during lymphocyte activation in immune synapse—during T lymphocyte activation (green), it interacts with an antigen-presenting cell (APC; blue) to recognize the antigen, presented by the APC. During this binding, there form three regions: central regions of supramolecular activation complex (cSMAC), peripheral SMAC (pSMAC), and distal SMAC (dSMAC). TCR/peptide–MHC interaction occurs in the cSMAC region and is required for the T-cell activation, whereas force-dependent integrin–ligand (LFA-1/ICAM1) interactions take place in the pSMAC region, which surrounds the inner cSMAC region. This results in the formation of focal adhesion complexes inside the lymphocyte at the immunological synapse. This integrin interaction at the pSMAC plays a crucial role in the co-stimulation of T-cell activation by forming adhesome enriched with talin bounded actin–myosin complex. Additionally, the interaction between TCR-antigen–MHC complexes in the cSMAC also occurs under force and forms catch bonds up to ∼10 pN (Huse, 2017).

Immune cells are known to migrate towards their destined site by rolling, and then it tethers and firmly adheres to the ECs, further transmigrating into the tissue by diapedesis (Figure 2). An example of precise spatiotemporal adhesion regulation under force is leukocyte rolling, which is mediated by selectins. It is plausible that shear force might be disruptive and impede leukocyte adhesion; however, it has been observed to be essential for optimal selectin-dependent adhesion. An AFM study has revealed that selectins form catch bonds with an optimum force of <20 pN (Marshall et al., 2003). Also, at a shear stress of >6 dyn/cm^2^ and pulling force of ∼35 pN per microvillus, leukocyte rolling is stabilized by the dynamic transition between slip and catch bonds. As immune cells tether to the ECs, a firm adhesion takes place through the integrin interaction with cell adhesion molecules (CAMs) on ECs. For example, T cell with the expressed integrin interacts with ICAM1 on the ECs. These integrin–CAM interactions are force-dependent and are allosterically strengthened within 10–30 pN of force (Sun et al., 2019). For example, in T cell, the expressed integrin LFA-1 gets activated either by activation of GPCRs on binding with chemokines or when auto-antigens are displayed by the antigen-presenting cells (APCs) to bind TCR, thus finally activating T cells in a mechanosensitive manner (Figure 3) (Savinov and Burn, 2010). Activation of TCR induces the interleukin-2 (IL-2)-induced T-cell kinase followed by activation of phospholipase C-γ1 (PLC-γ1) (Savinov and Burn, 2010). This PLC-γ1 induces a GEF Rap1 to form a complex with RAPL, eventually to induce the open conformation of αLβ2, or LFA-1, the most common integrin expressed in immune cells, to bind ICAM1. TCR activation also has the ability to phosphorylate GEF2 and induce Rap1 to ultimately change the conformation of αLβ2. Even GPCR activation leads to downstream signaling of PI3K, PLC, Rho, Ras, and MAPK-dependent pathways, which trigger the Rap1–RAPL complex to activate LFA-1 (Savinov et al., 2003; Kellermann et al., 2002; Amsen et al., 2000). These different modes of activation cause clustering of αLβ2/ICAM1 in the immune synapse, thus firmly adhering the T cells to the ECs even under shear stress (Figures 1, 2). Interestingly, cell rolling to adhesion at high shear stress helps T_H_ cells access the site of inflammation, which is significantly increased in the case of ADs (Skapenko et al., 2005; Bartholomäus et al., 2009). In the case of neutrophil motility, it was observed that these cells show integrin-dependent migration on surfaces as stiff as 12 kPa, whereas in less stiff surfaces (∼2 kPa), they show integrin-independent motility but exert a reduced traction force (Jannat et al., 2011b). Interestingly, during neutrophil transmigration, it has been shown to exert an immensely strong force of 60 nN per cell. Notably, the importance of mechanical threshold has also been noticed when B cells selectively internalized only high-affinity antigens, for optimally functioning as APC, before presenting it to CD4^+^ T cells (Huse, 2017). Interestingly, Tedford et al. proved that B cells adhere to the ECM very strongly at 3 dyn/cm^2^ of shear force in the murine model (Tedford et al., 2017). This binding is stabilized by LFA-1 interaction with ICAM1 and VCAM1. Due to a huge elevation of T-cell and B-cell functioning displayed by both systemic and organ-specific Ads, the role of NK cells becomes more prominent (Kucuksezer et al., 2021). Additionally, the guidance of NK cells towards specific tissue can be attributed to mechanical factors like tissue stiffness and cellular elasticity (Swaney et al., 2010). On the other hand, softer tissue (∼0.1–100 kPa) (Huang et al., 2012) causes talin polarization (which is defined as the localization or accumulation of talin at the cell–cell interface and is known to be integrin-dependent) in the interface of lymphocyte and target cell, forming unstable adhesion and lesser NK cell activation (Kupfer and Singer, 1989; Sedwick et al., 1999; Chakraborty et al., 2019; Friedman et al., 2021).

This suggests that T-cell and B-cell migration and homing (Matsumoto et al., 2017) can elevate autoimmunity in an integrin-dependent manner (Norman and Hickey, 2005). In addition to that, the success of anti-integrin antibodies in decreasing the effects of autoimmunity also supports the role of integrin in autoimmunity (Kawamoto et al., 20122012; Rath et al., 2018; Shannon and Mace, 2021). Furthermore, mechanical processes occurring in NK cells (Shannon and Mace, 2021), macrophages, and monocytes (Schittenhelm et al., 2017) also show their tissue residence during autoimmunity with the assistance of integrin. Therefore, considering these major immune cell processes, the role of mechanical force is inseparable from autoimmunity. More importantly, it confirms the obvious roles of mechanosensitive integrin in immune cell processes, causing the focal adhesome to regulate the biochemical and mechanical factors of immune cells.

### AUTOIMMUNE DISOEDERS REGULATED BY INTEGRIN

Autoimmunity is a multifactorial pathological abnormality that is due to factors ranging from abnormal genetics to environmental conditions. During AD progression, the self-reactive antibodies and self-antigens react in tissues and organs, creating inflammation and thus severe tissue damage (Jacobson et al., 1997; Eaton et al., 2007; Wang et al., 2015). However, the mere presence of potentially self-reacting lymphocytes does not cause pathological phenotype and is also found in healthy individuals. These lymphocytes produce the known natural autoantibodies required to remove the degraded self-antigens and keep foreign antigens in check to maintain homeostasis, such as rheumatoid factor and auto-nuclear antibody. This autoimmunity is called physiological autoimmunity where a normal individual does not show any pathological condition (Eaton et al., 2007). There are tolerance mechanisms that tightly regulate the production of auto-reactive lymphocytes in the body occurring in the thymus, bone marrow, and peripheral region before traveling through the circulating system. There is a positive selection of lymphocytes where self-antigens are displayed and made non-self-reactive. This is followed by negative selection and deletion of self-reactive lymphocytes. Even after negative selection, the autoreactive B cells are either deleted by clonal deletion or made inactive during peripheral anergy (Jacobson et al., 1997; Eaton et al., 2007; Xing and Hogquist, 2012; Wang et al., 2015). Only when these tolerance barriers are disrupted and self-reactive lymphocytes travel through the circulatory system to the site of inflammation or tissue displaying self-antigen does pathological autoimmunity develop (Xing and Hogquist, 2012). Some of these autoimmune disorders targeting different organs are discussed here and in Table 3, where several mechanically regulated immune cell processes take an active part through integrins and their ligands.

**TABLE 3:** Integrin and its ligands as a key contributor in the progression of autoimmune diseases

Disease	Integrins involved	Immune cells involved	Integrins role in autoimmune disease
Systemic lupus erythematosus (SLE)	Mac-1 (αMβ2)	B cells, neutrophils, and macrophages express high amount of αMβ2 (Rosetti and Mayadas, 2016)	• Mac-1 deficiency study induces hyper-immune response in SLE-prone mouse model (Kevil et al., 2004)
			• Non-synonymous mutation in Mac-1 gene ITGAM causes “R77H” mutation in the β propeller domain. This results in decreased catch-bond formation with ligand under shear force ranging from 0.19 to 0.42 dyn/cm^2^ and is directly associated with SLE. Most significant difference was observed at 0.32 dyn/cm^2^ (Rosetti et al., 2015)
			• Mac-1 promotes neutrophil accumulation in anti-glomerular basement nephritis by bearing the FcγR–IgG-mediated adhesion of neutrophils. Tang et al. (1997)
Crohn’s disease (CD)-	α4β1 (VLA‐4) and α4β7, αEβ7	NK cells, T and B lymphocytes, neutrophils	• CD is caused due to infiltration of leukocytes in the gastrointestinal tract with the help of α4β7-MadCAM1 (Erle et al. (1994), Newham et al., 1997)
			• Leukocytes can also be independently helped by α4β1/VCAM1 to transmigrate into the intestinal tract (Zundler et al., 2017)
			• αEβ7 expressing CD4^+^ T memory cells may be a major cause of inflammation due to CD, as αE^+^ T cells are known to destroy intestinal epithelial cells and are responsible for site-specific migration (El-Asady et al., 2005)
Ulcerative colitis (UC)-	αEβ7, α4β1 and α4β7	CD4^+^ T cells, T_H_1, and T_H_17 cells	• VCAM1 and MAdCAM1 are expressed highly in intestinal cells of UC patients, guiding α4β1 and α4β7 expressing cytotoxic and pro-inflammatory T cells into lamina propria
			• Inside lamina propria, T lymphocytes are retained by interaction between αEβ7 and E-cadherin of intestinal epithelia (El-Asady et al., 2005; Sandborn et al., 2005)
Type 1 autoimmune hepatitis	α4β7	CD4^+^ and CD8^+^ T cells, NK cells, γδT cells	• α4β7 integrin and CCR9 chemokine receptor-expressing T cells are generally not expressed much in liver cells. However, patients with IBD display MAdCAM1 and CCL25, ligands for α4β7 and CCR9, in their liver tissue. This causes the T lymphocytes, expressing α4β7 and CCR9, to migrate to liver from gut where any expression of auto-antigen either from gut or liver can cause immune response causing AIH (Eksteen et al., 2004; Adams and Eksteen, 2006; Oo et al., 2010)
Scleroderma	αVβ3, α5β1 and αVβ6 Mac-1 (αMβ2)	Macrophage, monocyte, B lymphocyte and T lymphocyte	• Fibrillin-1 is an ECM component that interacts with αVβ3, α5β1, and αVβ6 with its RGD-binding domain (Gerber et al., 2013)
			• Missense mutation of fibrillin-1 RGD domain, which interacts with integrin, can cause aggressive skin fibrosis (Gerber et al., 2013)
			• Disruptive cell–matrix interaction can cause upregulation of integrins, which can further be targeted as therapeutic agents (Gerber et al., 2013)
			• αM encoding gene ITGAM variant rs1143679 is linked with susceptibility towards systemic scleroderma (Carmona et al., 2011; Anaya et al., 2012)
			• MiR-150 regulates β3 integrin expression, which gets downregulated in lesions of systemic scleroderma (Carmona et al., 2011; Anaya et al., 2012)
			• Additionally, αVβ6-induced TGF-β expression can cause apoptosis resistance in fibroblasts (Gerber et al., 2013)
Psoriasis	α1β1 α6 integrin	T lymphocyte	• Inhibition of α1β1 to interact with collagen causes reduced accumulation of epidermal T cells. This has been observed with prevention of psoriasis (Conrad et al., 2007)
			• Integrity of laminin changes in psoriatic skin, causing insufficient interaction with α6 integrin (Conrad et al., 2007)
			• Hence, autoantibodies developed against α6 integrin cause the micro-wounds in skin (Gál et al., 2017)
Dermatomyositis	αVβ3	Monocytes, T lymphocytes, and B lymphocytes	• Neovascularization was increased in muscle biopsies of dermatomyositis juvenile patients (Nagaraju et al., 2006)
			• mRNA profiling showed upregulation of angiogenesis-related factors in dermatomyositis biopsies
			• Integrin αVβ3 assists in neovascularization, and its expression is higher in juvenile patients affected by dermatomyositis (Nagaraju et al., 2006)

### Type 1 Diabetes Mellitus

T1DM is one of the most prominent examples of AD, which results in the destruction of pancreatic islet β cells and requires lifelong treatment. Studies about human T1DM on nonobese diabetic (NOD) mouse models provided critical information about the roles played by T helper (T_H_) and T cytotoxic (T_C_) cells. The exposure of peptides, either post-translationally modified or insulin derived, to the autoreactive T cells in the pancreatic lymph node causes the generation of T memory cells against the pancreatic β cells (Jiang et al., 2016). Additionally, B cells also interact with the CD4^+^ T cells and cause autoantibody production against islet β cells (Schenkel et al., 2004; Huynh et al., 2011; Stroka and Aranda-Espinoza, 2011; Martinelli et al., 2014). Along with these T and B cells, neutrophils are also implicated in the instigation of insulitis and T1DM, as the reduction in blood neutrophil content was correlated with increased infiltration of neutrophils in the pancreatic islets leading to an occurrence of autoimmune T1DM in the NOD mouse. These studies have also revealed that the migration of lymphocytes from blood to secondary lymph nodes is one of the salient reasons for providing adaptive immunity as well as causing the autoimmune T1DM, and during this migration, mechanical force plays a crucial role (McIntyre et al., 2003; Mestas and Ley, 2008; Gaertner et al., 2022), as described previously. This directed migration is majorly assisted by the adhesive molecules expressed on the surface of immune cells and ECs (Campbell et al., 2003; Butcher and Picker, 1996; Ni et al., 2003; Myśliwiec et al., 1999; von Andrian and Mempel, 2003). Interestingly, during the early phase of T1DM, increased expression of adhesion molecules was observed, and inhibition of the same restricted the disease progression in the NOD mouse (Huang et al., 2005).

**FIGURE 4:**
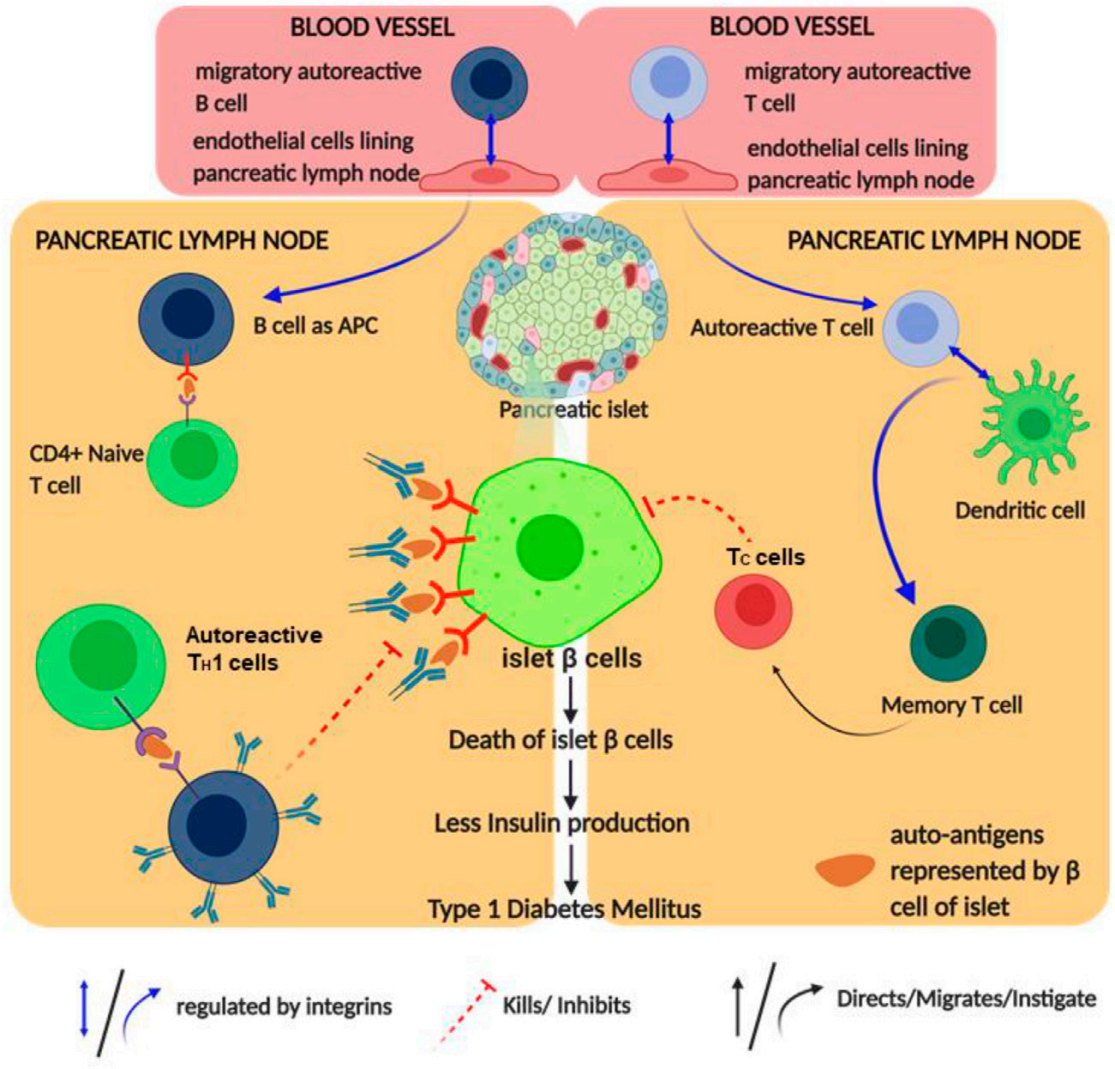
Schematic diagram of type 1 diabetes mellitus (T1DM) pathophysiology and its regulation by integrin: the figure provides a schematic diagram of how autoimmune diabetes mellitus causes and integrin regulates this disease. Blue arrows denote signaling/mechanism being regulated by integrin.

Different integrin interactions are known to exhibit biphasic force dependency, where the bond lifetime first increases with the force (known as catch bond), followed by a decrease in lifetime metrics with a further increase in force (known as slip bond) upon achieving the force maxima. This peak force is where the bond lifetime is the highest, and long-lifetime complex fractions are mostly observed. For firm adhesion, force-sensitive interactions of LFA-1, and Mac-1 with ICAM1, as well as between α5β1/FN, are indispensable for T cell and EC interactions (Sun et al., 2019). These integrin–ligand interactions have been reported to occur within defined force regimes. Such LFA-1/ICAM1 interaction is functional within 0–15 pN of force range, whereas α5β1/FN interaction is known to function within the 10–30 pN range (Kong et al., 2009; Chen et al., 2010). At <30 pN of force range, this bond formation has a prolonged lifetime due to a catch-bond formation; however, this prolonged lifetime decreases after >30 pN of force or below 20 pN, suggesting a force maxima at ∼30 pN of force. Other integrin interactions such as α4β1/VCAM1 and α4β7/MAdCAM1 are not mechanically characterized by force spectroscopy techniques (Baron et al., 1994; Hänninen et al., 1998). Interestingly, T cells interact with many APCs in lymph nodes, among which B cells are prime APCs that interact with T helper cells to initiate T1DM. Studies observed the B-cell role in the autoimmunity onset when these cells expressed adhesion molecules in different lymph nodes directly or indirectly linked to T1DM in a 3- to 4-week-old NOD mouse (Springer, 1995; Butcher and Picker, 1996; Xu et al., 2010). They observed that the α4, β7, and αLβ2 integrins were expressed by mostly all the B cells of the peripheral, pancreatic, and mesenteric lymph nodes. However, their expression did not correlate to their activity when observed in *in vivo* migration assay. Interestingly, inhibiting MAdCAM1 or α4β7 with specific mAbs reduces the B-cell migration into the pancreatic lymph node, thereby reducing the occurrence of T1DM (Xu et al., 2010). In an AFM study, the unbinding force of α4β7/MAdCAM1 interaction has been measured to be within 32–80 pN of force at a loading rate of ∼100 to ∼2,700 pN/s (Wang et al., 2018). However, inhibition of αLβ2, expressed as highly as α4, is unable to impede the B-cell migration effectively, and thus, a single integrin is not capable of deciding the migratory fate of the cell for causing the disease. Although the role of αLβ2 might not be as important in the B-cell migration and causation of T1DM, its significance cannot be ruled out in the pathogenesis and progression of T1DM (Savinov and Burn, 2010; Xu et al., 2010; Huang et al., 2016). Early studies on LFA-1 in T1DM causation showed that expression of its αL subunit decreased and αLβ2 expression on monocyte was normal, suggesting LFA-1 as not indispensable for the pathology of T1DM (Martin et al., 1991). Nevertheless, knocking out any of the subunits of αLβ2 integrin prevented insulitis even in the advanced diabetic stage in NOD mice. Specifically, eliminating the β subunit restricted the T-cell adhesion to ECs, whereas the absence of α subunit inhibited it, speculating the biochemical regulatory role of integrin by transducing the force (Huang et al., 2005). Furthermore, studies have found a very high expression correlation in monocytes, and its counter ligand ICAM1, along with islet cell-based autoantibody titer (Martin et al., 1991; Myśliwiec et al., 1999; Bertry-Coussot et al., 2002). Due to the constitutive expression of LFA-1 in different kinds of immune cells, it becomes a target for proteins to control the pathogenesis of T1DM. Indeed, inactivating the LFA-1 with its mAbs caused the delayed occurrence of T1DM, blocking the disease pathology. Specifically, a cyclic peptide cLAB.L has been engineered to prevent the D1 domain of ICAM1 on ECs with αI domain of T cell LFA-1, suggesting the regulatory effect of its αI domain on T-cell adhesion to the microvascular endothelium (Huang et al., 2005). In addition, even in the presence of other adhesion molecules like α4β1 and VCAM1, this T-cell interaction with microvascular endothelium is critically dependent on the αI domain, reconciling the importance of LFA-1 directly in the causation of T1DM (Huang et al., 2005). Since the progression of T1DM is crucially regulated by mechanically regulated immune cell processes like lymphocyte migration, adhesion, and interactions, integrin adhesome proves the integral role of mechanical force in this disease progression (Figure 4).

### Rheumatoid Arthritis

RA is a highly aggressive and complex inflammatory disorder, affecting majorly the synovial joints of hands and feet that lead to joint destruction, chronic disability, and poor life quality (Smolen et al., 2007; Smolen et al., 2018). The disease onsets with non-organ specific autoantibodies, produced as a consequence of this disease, cause further inflammation of other organs, leading to serious cardiovascular, pulmonary, or skeletal complications (Smolen and Steiner, 2003; Firestein, 2005; Smolen et al., 2007; Smolen et al., 2016; Firestein and McInnes, 2017; Smolen et al., 2018). The HLA-DRB1 locus of the MHC complex was found to be associated with RA, by assisting antigen presentation to T cells during the induction of autoimmunity. Studies with SKG mice (murine model for understanding RA pathogenesis) provided the link of autoreactive T-cell activation, selection, and its interaction with innate and adaptive immune cells, resulting in the production of autoantibodies and RA onset (Sakaguchi et al., 2003; Firestein, 2004; Firestein, 2005). Additionally, while treating the RA patients with rituximab, a chimeric mAb targeting CD20 on B cells, the role of B lymphocyte also became prominent in RA. Due to the B-cell abundance in synovial fluids of inflamed joints, rituximab can be a therapeutic agent for RA treatment (Dörner and Burmester, 2003; Edwards et al., 2004; Tsokos, 2004). Other cells such as fibroblast-like synoviocytes and chondrocytes interact with T cells, accelerating the joint destruction in RA patients. Direct or indirect production of IL-17 cytokine by T-cell simulation causes fibroblasts, T cells, or macrophages to infiltrate the inflamed joints. It has also been observed that IL-17 induces MMP production, which changes the bone metabolism towards osteoclastogenesis, leading to bone resorption (Chabaud et al., 1999; Koshy, 2002; Stamp et al., 2004). These studies highlight the importance of self-reactive T cells and their interacting cells, playing a significant role in RA.

Highly proliferative synovial fibroblasts (SFs) line the synovial lining of joints and act as a major player in severe cartilage and bone destruction during RA progression (Mellado et al., 2015). T_H_1 cells activate macrophage, SF, and ECs in the joints, creating an inflammatory niche by the release of cytokines, matrix-degrading enzymes, and overexpressing integrin-like adhesion molecules (Sweeney and Firestein, 2004). These attachments with ECM proteins are controlled by the expression of ICAM1 and αLβ2 integrin, which have been reported to optimally interact under 10–15 pN of applied load (Chen et al., 2010). The enriched presence of IL-1β in the synovial tissue of RA increases the ICAM1 expression, in the proinflammatory niche of the RA, which is the major interacting partner of αLβ2 integrin (Lowin and Straub, 2011). As αLβ2 is expressed in the majority of immune cells and is required during the guidance of leukocytes to the synovial tissues, it majorly contributes to the development of inflammation (Lowin and Straub, 2011). Additionally, the expression of a laminin-binding integrin-α6β1 in the synovial lining provides an interesting mechanical insight into the causation of RA, as laminin and integrin interactions are thought to be mechanically regulated (Takizawa et al., 2017). By contrast, the expression of α4β1 is very high in the synovial tissue T cells, if compared to that residing on the tissue lining (Hyun et al., 2009). Since VCAM1 expression is very high on RA ECs, it attaches to the α4β1 integrin of T lymphocytes and assists them in the migration to the site of inflammation (Hyun et al., 2009). Zhang et al. observed that an individual α4β1/VCAM1 complex may experience <50 pN of force during the leukocyte activation by AFM spectroscopy with a loading rate of 100–100,000 pN/s. At this force range, the interaction is capable of forming a strong adhesion. Interestingly, during the rolling process, this α4β1/VCAM1 could work within 50–250 pN; however, the dissociation rate at this regime becomes less force-dependent and can exhibit mechanics similar to those of the αLβ2/ICAM1 complex (Zhang et al., 2004). Even in collagen-induced arthritis, α4β1 antagonists have shown prevention of inflammation and MMP production (Lowin and Straub, 2011). Synovial tissue resident cells express α5β1 and αvβ3 integrins, which exhibit force-dependent interactions with their respective ligands like fibronectin, vitronectin, and bone sialoprotein, within a range of 0.1 pN to tens of pN (Li et al., 2003; Sun et al., 2005; Roca-Cusachs et al., 2009; Chang et al., 2016).

**FIGURE 5:**
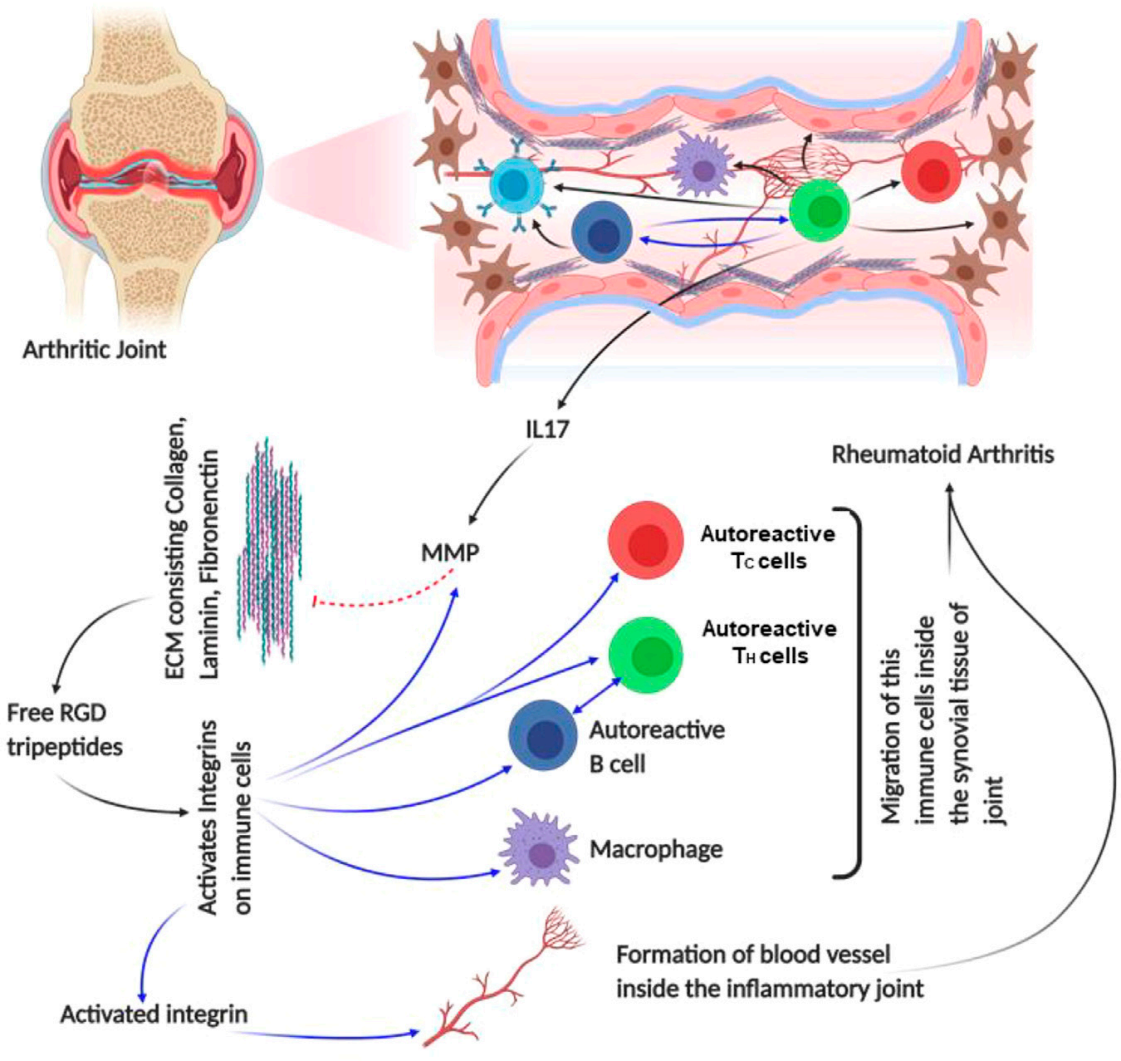
Schematic diagram of rheumatoid arthritis pathophysiology: the figure provides a schematic diagram of how rheumatoid arthritis develops and what points of this disease are regulated by integrin. Blue arrows denote signaling/mechanism being regulated by integrin.

The function of αvβ3 in RA inflammatory tissue remains unclear, as it is reported to assist angiogenesis while interacting with vascular endothelial growth factor receptor 2 (VEGFR2) during tumor progression (Wilder, 2002; Alghisi et al., 2009). As angiogenesis is also required for proper RA, it has been observed that an αv antagonist prohibits the growth of blood vessels in the inflamed region (Wilder, 2002; Alghisi et al., 2009). αvβ3 increases the bone resorptive capability of the osteoclast cells by initiating FAK and c-Src signaling, which helps in transducing the force sensed through integrin molecule (Wilder, 2002; Alghisi et al., 2009; Lowin and Straub, 2011). In the inflammatory tissue, β1 and β3 subunits are predominantly expressed, which are known to bind diverse interacting partners like fibronectin, laminin, collagen, and vitronectin in synovial tissue (Charo et al., 1990; Davis et al., 1990; Pankov and Yamada, 2002; Hoberg et al., 2006). Degradation of the collagens by MMPs frees up the RGD peptides, which go on to activate several integrins like αvβ3, α5β1, or αIIbβ3 (Davis, 1992). However, primary integrins getting activated by RGD peptides in arthritic condition are α1β1 and α2β1, which bind to collagen. In osteoarthritis, α1 is found on the blood vessels of arthritic joints and synovial lining, but in the presence of cortisol, the SFs also show massive expression of this adhesive subunit (Rubio et al., 1995; Lowin et al., 2009; Lowin and Straub, 2011). Thus, the inflammatory milieu of RA assists in the overexpression of α1 integrin similar to the α5, otherwise induced by cortisol (Takahashi et al., 1992). VEGF in synovial tissue upregulates α1 integrin, a prime regulator of angiogenesis required for continuous progression of RA (Senger et al., 1997). Thus, the inhibition of α1 and collagen will prevent the formation of new blood vessels, providing a therapeutic target for RA. In a murine model of anti-collagen II antibody-induced arthritis, the prevention of α1 integrin has shown decreased cartilage degradation and leukocyte infiltration. Similar to collagen, laminin ligands—α3β1, α6β1, α7β1, and α6β4—assist in cell adhesion and migration. Especially, α3β1 in the synovial tissue and α6β1 in the synovial lining fibroblasts are highly expressed, leading to the upregulation of laminin. This eventually increases the expression of MMP3 and MMP10 and activates integrin (Davis, 1992; Hoberg et al., 2006). In addition, inflammatory fibrous tissue in RA has been observed to infiltrate with macrophage, and T and B lymphocytes, which predominantly express α2β1 integrin on their surfaces. However, antigen-induced arthritis (AIA) mice lacking in α2β1 integrin show decreased MMP3 expression due to anomaly in ERK activation in both sera and fibroblast-like synoviocytes (Davis, 1992; Pfaff et al., 1993; de Fougerolles et al., 2000; Wilder, 2002; Peters et al., 2012). These findings suggest that different β1 integrins enhance the inflammatory cartilage degradation by different means, ranging from fibroblast proliferation to MMP expression. Similarly, fibronectin-coated synovial cells attract lymphocytes expressing α4β1 and α5β1 integrin where the α5 integrin subunit is largely expressed in the synovial tissues as well as the cells lining it (Davis et al., 1990; Davis, 1992; Hoberg et al., 2006; Lowin and Straub, 2011). These examples of different integrins along with their ligands, interacting in a force-dependent manner, define the regulatory role that integrin plays in the cause and progression of RA. Additionally, by the application of antagonists designed against these adhesive molecules, partial prevention or onset of the disease might be delayed (Figure 5).

### Multiple Sclerosis

MS is a demyelinating, inflammatory disorder of the central nervous system (CNS), affecting the global population (Zhang et al., 2020). Two-thirds of the patients show a relapse of the disease where inflammatory lesions with B cells, T cells, or macrophages are observed in the white matter, and the axons and neurons are subject to inflammation or degradation in the gray matter (Steinman, 2009; Lee-Chang et al., 2011; Miljković and Spasojević, 2013). In the majority of the MS studies, researchers have used an EAE mouse model to understand this pathological abnormality, as this model displays both progressive and relapsing–remitting types of MS. In EAE mouse, encephalitogenic leukocytes cross the blood–brain barrier and cause damage in neuronal and axonal myelin sheaths, which has revealed the hyperactivity and release of auto-reactive T cells in the progression of MS (Handel et al., 2010; Mkhikian et al., 2011; Miljković and Spasojević, 2013). In addition to cytotoxic T cells, helper T cell subsets T_H_1 and T_H_17 are the most autoreactive T cells responsible for CNS damage (Petermann and Korn, 2011). Consequently, these autoreactive T_H_ cells recruit immune cells like macrophages, neutrophils, and B cells to attack the cells displaying self-antigens, making them autoreactive as well (Miljković and Spasojević, 2013). In CNS, CD27^+^ B memory cells are a major source of producing antibodies, while other B cells are involved in the production of cytokines such as IFNγ or IL-12-like inflammatory substances, making the migration of B cells across CNS endothelia a major reason in the initiation of MS (Lee-Chang et al., 2011).

**FIGURE 6:**
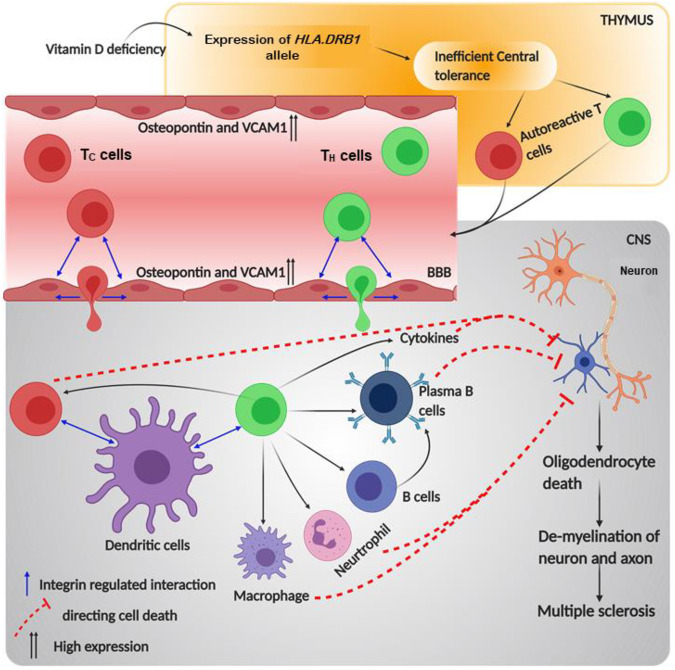
Schematic diagram of multiple sclerosis pathophysiology and role of integrin in its progression: the figure provides a schematic diagram of how multiple sclerosis develops and what points of this disease are regulated by integrin. Blue arrows denote signaling or mechanism being regulated by integrins.

Microarray analysis on the EAE pathogenesis has provided substantial insight on molecular players that regulate the migration of T or B lymphocytes and other autoimmune responsive cells into the CNS (Chabas et al., 2001). Notably, in MS murine model, the paralysis and abnormal conduction through nerve decrease due to intravenous treatment with anti-α4 and anti-β1 molecules by blocking the T cell binding to inflamed brain endothelium (Yednock et al., 1992; Baron et al., 1993; Steinman, 2005). In encephalitogenic cells expressing α4β1 integrin, treatment with anti-β7 mAbs showed a partial remission along with a diminished EAE. This was due to the possible involvement of either α4β7 or αEβ7 integrins in MS pathogenesis, as β7 subunit couples with these two α subunits. Interestingly, the application of both anti-α4 mAbs and anti-β7 mAbs brought complete remission to the encephalitogenic cells. Additionally, it decreased the complete remission period to 4–5 days from 50 days when otherwise treated with only anti-α4 mAbs. However, the application of anti-β7 mAbs did not reduce the progression of the MS; its importance was noticed when β7 knock-out T cells failed to proliferate as control (Kanwar et al., 2000). These experiments reconciled the role of α4 and β7 integrin subunits in the causation of MS. Additionally, it was found that after complete remission in antibody-treated EAE mice, integrin ligands like MAdCAM, VCAM1, and ICAM1 proteins were either not expressed or less expressed if compared to the high expression in severe disease conditions (Kanwar et al., 2000). Interestingly, it is already known that ligand molecules expressed on APCs like DCs are ligands of α4β7 and αEβ7 on T cells and are required for co-stimulation (Szabo et al., 1997; Lehnert et al., 1998; Berg et al., 1999; Ebner et al., 2004). Transportation of the autoreactive T cells occurs due to the expression of VCAM1 and osteopontin in the inflamed brain tissue. Osteopontin, an N-linked glycoprotein, is expressed majorly on the inflamed EC of the blood–brain barrier and binds to α4β1 integrin (Fisher et al., 2001). Thus, T cells expressing α4β1 bind to the counter ligands of ECs and diapedase through the endothelia. Once these T cells get inside the brain, they encounter self-antigen displayed by the APCs and release a plethora of cytokines. These cytokines damage the oligodendrocytes, which are responsible for myelin production. In addition, activation of B cells by T_H_ cells produces antibodies against myelin, creating a proper inflammatory niche in the CNS (Steinman, 2009). Therefore, integrins like α4β7, αEβ7, and α4β1 and their respective ligands are responsible for the progression and development of MS by regulating the processes of immune cells (Figure 6).

### Vitiligo

Vitiligo is an acquired disorder of skin depigmentation that is progressive in nature, causing hypomelanosis of the skin and hair due to the total absence of melanocytes. This causes depigmented patches all over the body, affecting the physiological and psychological health of almost 0.5%–2% of the world population (Ongenae et al., 2005; Ramakrishna and Rajni, 2014; Iannella et al., 2016; Salman et al., 2016; Su et al., 2019). Vitiligo was unsurprisingly considered to be an autoimmune disorder involving several humoral and cellular components of the adaptive and innate immune systems. This was based on a strong correlation of being associated with other ADs such as pernicious anemia, T1DM, myasthenia gravis, psoriasis, Addison’s disease, and Grave’s disease (Gauthier et al., 2003; YAGHOOBI et al., 2011). Indeed, genes related to autoimmune susceptibilities such as HLA, PTPN22, CTLN4, and NALP1 were reported to be involved in vitiligo too (Badri et al., 1993). Additionally, similarities with other ADs like the chronic relapse and remission, circulating anti-melanocyte antibodies, and response to immunosuppressive treatments were observed for vitiligo (Farrokhi et al., 2005; Glassman, 2011). Moreover, the periphery of vitiligo lesions shows sparse infiltration of CD8^+^ T cells, a key characteristic of autoimmune disorder (Pichler et al., 2009; YAGHOOBI et al., 2011). Additionally, a sharp increase in the ratio of T_H_ to T_C_ cells was observed in vitiligo patients; however, the B cell role was not observed directly in tissues. The memory T cells express CLA, which is known to bind E-selectin of dermal ECs (Glassman, 2011; YAGHOOBI et al., 2011). Interestingly, CLA^+^ T cells that clustered around disappearing melanocytes are cytotoxic, i.e., are positive for both granzyme B and perforin (Glassman, 2011; YAGHOOBI et al., 2011). Notably, the release of these enzymes is remarkably regulated by force through integrin adhesome (Keefe et al., 2005; Thiery et al., 2011). Moreover, the release of IFNγ and CXCL10 forms the CD8^+^ T cells, as observed in the mouse model of vitiligo, which proved how T_C_ cells are directed towards lesion sites in the epidermis (Birlea et al., 2017). Thus, the role of T_C_ cells in vitiligo pathogenesis becomes quite prominent due to its capability of attacking the automelanocytes. Although the complete mechanism of vitiligo remains elusive, several theories have been postulated to describe its plausible causation. Among these, the theory of “melanocytorrhagy” majorly focuses on the depigmentation of vitiligo patches, due to the detachment of melanocytes in the presence of mechanical stress (Gauthier et al., 2003; YAGHOOBI et al., 2011).

**FIGURE 7:**
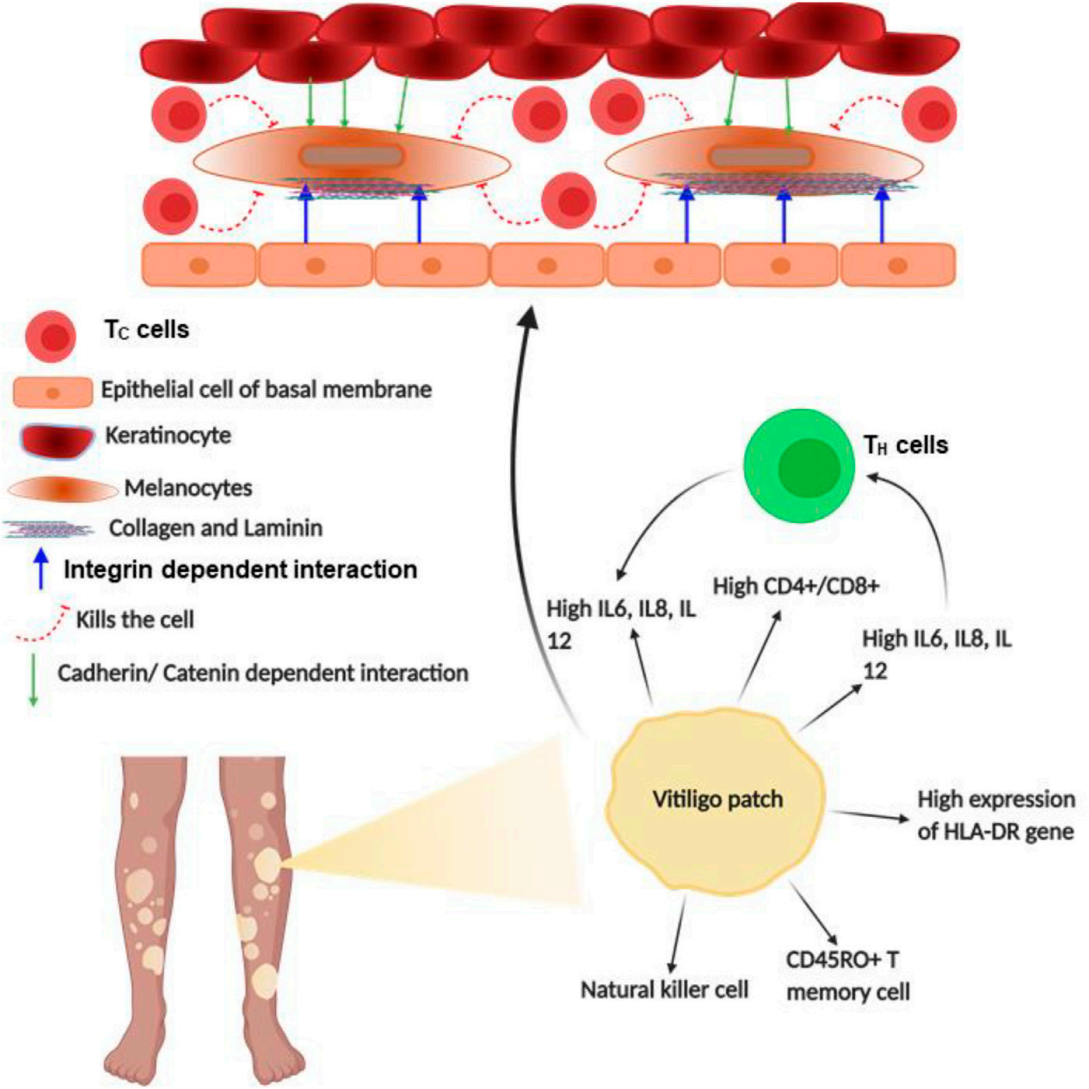
Schematic diagram of vitiligo pathophysiology: the figure provides a schematic diagram of how vitiligo develops and what points of this disease are regulated by integrin. Blue arrows denote signaling or mechanism being regulated by integrins.

According to the “melanocytorrhagy” theory, the decrease in melanocyte number occurs not only due to T_C_ cells but also due to decreased adhesion of it from the keratinocyte of the basal membrane, allowing it to migrate and separate from the epidermis, resulting in vitiligo patches. The cell–cell interaction regulating paracrine and adhesive molecules from keratinocytes is also responsible for tuning melanocyte decoupling, migration, and recoupling elsewhere (Ezzedine et al., 2015; Birlea et al., 2017; Su et al., 2019). Thus, the adhesion molecules' role gradually becomes clear in the causation of vitiligo, as the adhesion and migration of melanocytes are regulated majorly by these adhesive molecules. Recent discoveries also found the role of adhesive molecules in regulating the initiation and pigmentation procedure in vitiligo (Reichert Faria et al., 2017; Su et al., 2019). However, among these adhesive molecules, cadherin and catenin are major proteins that form the intercellular junctions between the keratinocyte and the melanocyte, whereas the melanocyte connects to the basal membrane through the expressed integrin and the corresponding ligands especially collagen and laminin, which interestingly are regulated in a force-dependent manner (Reichert Faria et al., 2017; Su et al., 2019). These adhesive molecules regulate the melanocytes' connection with keratinocytes and basal membrane. Interestingly, it was hypothesized that miR-9, a neural crest cell micro-RNA inhibitor, might have a regulatory role in melanocytes of vitiligo lesions. This regulatory miRNA reduces different adhesive molecules such as β catenin, E-cadherin, laminin, collagen IV, and β1 integrin during tumor progression in neural crest cells (Su et al., 2019). Similarly, this effect was also observed for melanocytes, and the reduction in adhesion molecules caused lesser decoupling of melanocytes from the epidermis or adjacent keratinocytes. Particularly for PIG1 melanocyte cells and HaCaT keratinocyte cells, it was observed that less migration of PIG1 occurred from the HaCaT cells due to miR-9 treatment. This shows how β1 integrin and its ligands (collagen and laminin) are extensively involved in the decoupling–migration–recoupling mechanisms of melanocytes leading to pigmentation anomaly. Additionally, ligands of β2 integrin like ICAM1 and VCAM1 have also shown constant expression on vitiligo melanocytes (Su et al., 2019). Moreover, constitutive expression of ICAM1 has been observed to be linked with the abnormal immune response of melanocytes (Ezzedine et al., 2015; Birlea et al., 2017; Reichert Faria et al., 2017; Su et al., 2019). Additionally, another group showed expression of ICAM1 in perilesional melanocytes of active patches of vitiligo. Since β2 integrin in neutrophils has been found to interact with ICAM1 in its high-affinity bent-open conformation at ∼6 dyn/cm^2^, it also generates a possibility of force-dependent interaction for the melanocytes (Glassman, 2011). Moreover, during re-pigmentation, there are subsequent changes in integrin expressions, which otherwise showed no observable difference in lesioned, non-lesioned, or normal skin (Birlea et al., 2017; Reichert Faria et al., 2017; Su et al., 2019). This proves that integrin and its ligands are key players in the mechanically regulated melanocyte adhesion as well as the detachment during the pathogenesis of vitiligo, making it a proper therapeutic target (Figure 7).

## Outlook

**FIGURE 8:**
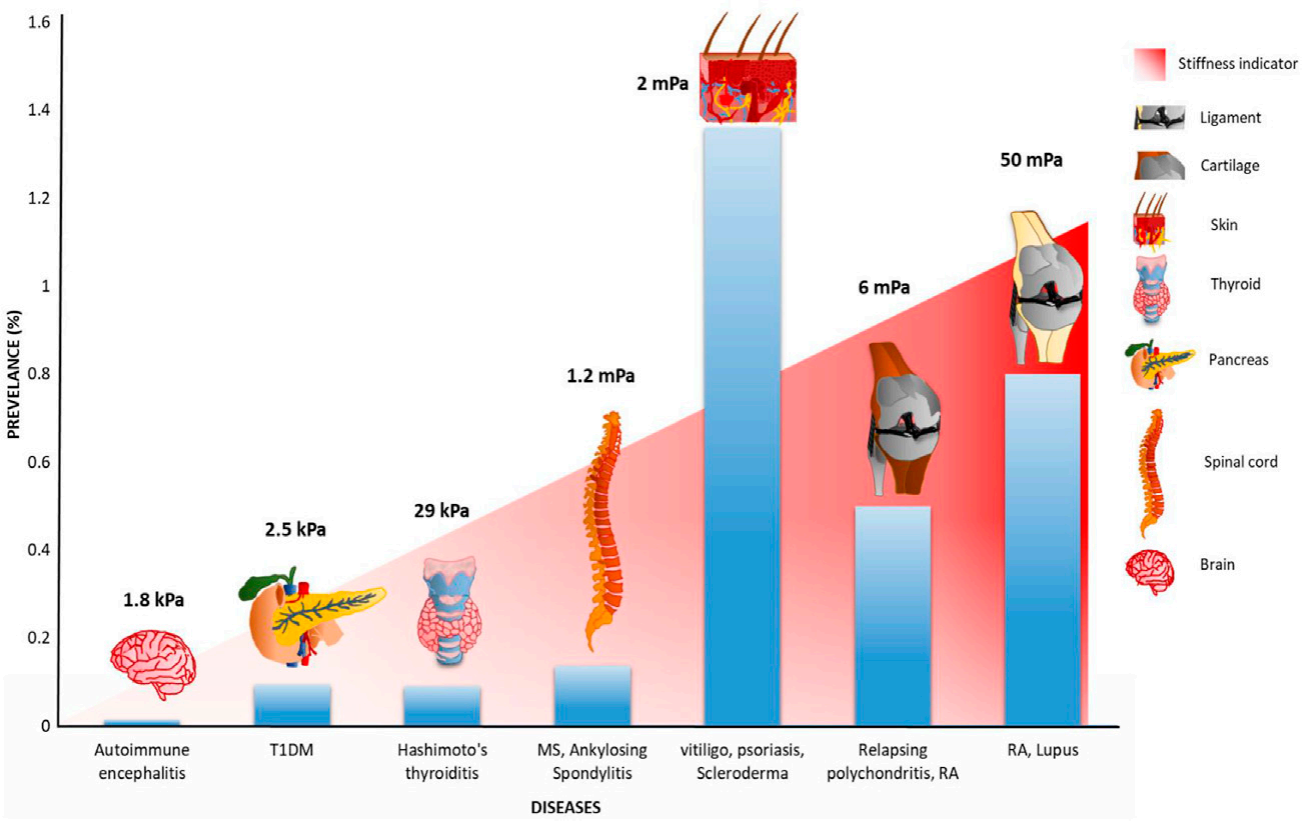
Plausible correlation between the worldwide prevalence of different autoimmune disorders with organ stiffness: the prevalence percentage of different autoimmune disorders affecting differentially stiff organs has been illustrated. Autoimmune diseases range from autoimmune encephalitis, type 1 diabetes mellitus (T1DM), and autoimmune thyroiditis, which affects softer tissues like the brain, pancreas, and thyroid, respectively, to vitiligo, psoriasis, multiple sclerosis, and lupus scleroderma, which affect stiffer or hard tissues, have been considered for this study. As the figure depicts, the trend of autoimmune disease prevalence shows a positive correlation with the different organ stiffness. For example, in the case of autoimmune thyroiditis, the worldwide prevalence rate is approximately 0.1%, which majorly affects the thyroid with tissue stiffness of 29 kPa (Guimarães et al., 2020), whereas, with lupus, which affects ligament (>5 MPa), the prevalence rate increases to 0.8%. Autoimmune encephalitis affected the brain; type 1 diabetes mellitus affected the pancreas; Hashimoto’s thyroiditis affected the thyroid; multiple sclerosis and ankylosing spondylitis affected the spinal cord; vitiligo, psoriasis, and scleroderma affected the skin; relapsing polychondritis and rheumatoid arthritis affected the cartilage; rheumatoid arthritis and lupus affected the ligament (Chopra et al., 2013; Baldini et al., 2017; Resende de Paiva et al., 2017; Dubey et al., 2018; Parisi et al., 2020; Siebert, Raj, Tsoukas; Walton et al., 2020; Almutairi et al., 2021; Barber et al., 2021).

Mechanical force plays an integral role in regulating diverse cellular processes ranging from protein translation, translocation to cell adhesion, and migration (Wruck et al., 2017; Vicente-Manzanares et al., 2009; Haldar et al., 2017; Goldman et al., 2015). The recent development of force spectroscopy technologies has provided an access to measure the force sensed by mechanosensitive proteins of immune cells. Furthermore, studies on immune cell mechanics provided information on the regulatory roles of force in different cellular processes (Franck et al., 2011; Benoit et al., 2000; O'Donoghue et al., 2013; Kienberger et al., 2005; Zhang et al., 2020; Hosseini et al., 2009; Pageon et al., 2018; Vorselen et al., 2020). Immune cell interaction, activation, and signaling that occurred during their migration process suggest the plausible role of mechanical force at the cellular level. Though mechanical force has been reported to play a key role in immune system functioning, how its perturbation drives autoimmunity progression has not been studied yet (Natkanski et al., 2013; Hui et al., 2015; Basu et al., 2016; Lämmermann et al., 2008). It is well known that matrix or tissue stiffness is a critical factor in different pathological conditions such as cancer metastasis (Bauer et al., 2020). However, its plausible role in the development of ADs is not studied much and remains elusive. Additionally, a change in substrate stiffness results in heritable epigenetic modifications, which in turn causes ADs by regulating gene expression (Janmey et al., 2020; Mazzone et al., 2019). A study by McCullough et al. showed an empirical relevance of stiffness in myositis disease, where reduction in muscle stiffness is correlated with the disease progression (McCullough et al., 2011). Recently, an AFM study has shown that autoimmune insulitis is governed by the changes in islets stiffness due to hyaluronan reduction in ECM (Nagy et al., 2018). A clinical study by Yada et al. has speculated that liver stiffness could be a critical factor in autoimmune hepatitis; however, further studies are required to reconcile the role of stiffness (Czaja, 2014). Arterial stiffness has also been reported as a factor for systemic vasculitis (Booth et al., 2004). This stiffness-mediated autoimmune progression has not been demonstrated at the cellular level; however, an AFM-TEM study has shown that mechanical disruption of collagen alters the matrix composition, which in turn changes the mechanical stability of the ECM network in RA (Antipova and Orgel, 2012; Maldonado and Nam, 2013; Poole et al., 2002). Overall, this varied stiffness results in two phenomena: either it detaches from the soft matrix, or it adheres too much to the stiffened matrix (Janmey et al., 2020). Matrix stiffness-regulated MMP activity has been reported in cancer-associated liver fibrosis; and thus, it is also plausible that it plays a critical role in liver fibrosis condition in type 1 autoimmune hepatitis (Lachowski et al., 2019). In response to the stiffened matrix, cells use its invadopodia to degrade the stiffened matrix using secretory MMPs (Janmey et al., 2020). MMP involvement has also been reviewed by Ram et al. (2006). This degradation helps the cells to migrate through the stiffer tissues. However, these degraded ECM peptides can act as major ligands in integrin activation, causing anomalies in mechanotransduction events. For example, we have discussed in the case of RA that MMP degrades collagen and that laminin frees the RGD peptide, which activates integrins, finally causing severe autoimmune disorder (Charo et al., 1990; Hoberg et al., 2006; Pankov and Yamada, 2002; Davis et al., 1990; Davis, 1992). Hence, modulated-tissue stiffness (or surrounding substrate stiffness) assists in the development of pathological conditions, providing insight on how tissue stiffness of different organs could result in ADs. Interestingly, fibulin-5 has been reported to be increased in skin tissues of systemic scleroderma patients. Indeed, loss of fibulin-5 prevents the inflammation and fibrotic phenotype in an animal model, which is a prominent pathological feature in any autoimmune disorder. The same study has also shown that a small change in matrix stiffness (2.5 times) upregulates chemokine expressions, which is also a linchpin factor in autoimmune disorders (Nakasaki et al., 2015; Karin, 2018). Additionally, integrin-modulating therapy has also been shown to be effective in scleroderma-associated fibrosis conditions. Integrin therapy also restores the skin stiffness in the patient sample (Gerber et al., 2013). This suggests integrin be used as a therapeutic agent, which directly connects the extracellular region and could be a factor for matrix stiffening in different autoimmune disorders. Other autoimmune disorders could also be speculated to be substrate or tissue stiffness-dependent. Autoimmune encephalitis, T1DM, or autoimmune thyroiditis, which specifically target the brain, pancreas, and thyroid, respectively, have a prevalence percentage much lesser than 0.1% (Dubey et al., 2018; Baldini et al., 2017; Resende de Paiva et al., 2017). However, on stiffer tissues such as the skin, spinal cord, or cartilage, the disease prevalence rate increases well beyond 0.1% and provides a correlation that stiffer tissues are more affected by ADs (Baldini et al., 2017; Parisi et al., 2020; Siebert et al., 2016; Walton et al., 2020; Almutairi et al., 2021; Chopra et al., 2013; Barber et al., 2021). Data introspection suggests that the prevalence of ADs on softer tissues (<100 kPa) is much lower as compared to stiffer tissues or hard tissues (Figure 8). Recently, the mechanical strain has been reported to play a regulatory role in arthritic inflammation and tissue damage (Cambré et al., 2018). This suggests force as a linchpin regulator during the causation of AD during migration and activation of immune cells.

It is well known that integrin regulates physical and biochemical processes during autoimmune disorders; however, integrin mechanics have not been clearly defined during autoimmunity. Throughout this review, we have shown that different integrin subunits are mechanically involved in the pathophysiology of ADs. In the majority of these disorders, integrin along with its ligands are regulated by bidirectional force transmission through an integrin–talin–actin mechanical linkage. This regulates the migration and activation of the self-reactive lymphocytes in the site where self-antigen is detected. Due to the indispensable role of integrin in mediating ADs, it has been suggested as a potential therapeutic target. Anti-integrin antibodies and small molecules, targeting specific integrin subtypes, reduce the integrin-mediated immune activity in pronounced inflammatory conditions. Natalizumab, vedolizumab, and lifitegrast are well-known anti-integrin therapeutics used in AD treatments such as Crohn’s disease and MS (Park and Jeen, 2018; Slack et al., 2022). Unlike broad immune inhibitors such as corticosteroids and TNF inhibitors, anti-integrin therapeutics possess reduced risk factors (Sattler et al., 2021). Glucocorticoids, a class of corticosteroids, can act against autoimmune conditions by interfering with the function of L-selectin and LFA-1, thereby reducing the neutrophil *trans*-endothelial migration (Filep et al., 1997). Similarly, dexamethasone increases αvβ3 expression in cells; however, using these drugs has severe dose-dependent toxicity (Saag et al., 1994; Huscher et al., 2009; Davis et al., 2011; Fan et al., 2012). Despite concerns regarding the use of small-molecule integrin inhibitors due to their less specificity and off-target effects, they are much safer to use because of their efficacy and specificity (Millard et al., 2011). However, anti-TNF drugs along with vedolizumab have shown promising effect in vedolizumab refractory patients (Rath et al., 2018). Moreover, considering the systematic complication of AD pathophysiology, experiments can also be performed with anti-integrin therapy accompanied with specific signaling regulators to increase treatment efficiency. Vedolizumab, an anti-α4β7 integrin antibody, is approved for the treatment of inflammatory bowel disease (IBD), an autoimmune disorder. This is known to inhibit the adhesion of leukocytes to the endothelium of the gastrointestinal tract, thereby impeding the interaction of α4β7/MadCAM-1 (Dotan et al., 2020). In a recent study, Rath et al. have shown through transcriptome analysis that vedolizumab reduces the adhesion and diapedesis of both granulocytes and agranulocytes (Rath et al., 2018). Similarly, natalizumab, generated against specific α4 integrin, is used for the treatment of EAE mouse and humans MS models (Kerfoot et al., 2006; Brandstadter and Katz Sand, 2017). This drug is shown to disturb the ability of leukocytes to transmigrate through the blood–brain barrier. Similarly, a small molecule named lifitegrast inhibits LFA-1–ICAM1 interaction to decrease lymphocyte migration and adhesion to the endothelial wall, acting as a potential drug for autoimmune dry eye disease (Perez et al., 2016). This suggests that these anti-integrin drugs are disrupting the force-dependent integrin interactions with their ligands, thereby interfering with integrin-dependent immune cell activities.

Currently, ssDNA and RNA have been designed as aptamers against the integrin α4 subunits to be used as therapeutics against MS (Kouhpayeh et al., 2019). Additionally, in RA, targeting integrin ligands like osteopontin by M5 antibody and antibody against β1 is under clinical trial (Yamamoto et al., 2003; Smolen et al., 2018). UVB-based therapy, for treating vitiligo lesions, targets β1 integrin and E-cadherin-like adhesive molecules in melanocytes to assist them to migrate towards the keratinocytes, thus re-pigmenting the white lesions (Su et al., 2019). These different mechanical roles of integrin in autoimmune disorders establish the importance of its mechanics involved in autoimmunity, which in turn could be a critical factor for designing the integrin-associated therapeutic targets. This information provides an insight into mechanical force playing a crucial role in autoimmunity, which has not been defined yet; however, the prevalence data suggest such a trend. Since integrin is regulated by force, mAbs designed against integrin could precisely tune its force-sensing ability. Additionally, the progression and effect of ADs on the target organs also depend on the elasticity of the ECM of those organs. This elasticity range can vary from as low as 50 Pa in the blood tissue to a very high value of 5,000–6,000 kPa in cartilage ECM (Guimarães et al., 2020). This broad range of elasticity will lead to different immune cell adhesion or migration in separate organs, demonstrating different destructive effects. Unfortunately, the lack of enough data is an obstacle in understanding these phenomena. Additionally, it is well known that integrin majorly regulates the migration of immune cells on stiffer surfaces, as observed in the case of neutrophil migration (Jannat et al., 2011b). Hence, we can correlate the fact that the AD causative immune cells show their mechanically regulated processes majorly through integrin-dependent adhesome. Moreover, not much is known about the role played by mechanosensitive proteins like talin, actin, and myosin of the integrin adhesome complex in autoimmune disorder, as the force is transmitted through them. Therefore, targeting these mechanosensitive proteins and regulating their biochemical and force sensing capability can provide a new horizon in autoimmune therapy. Thus, understanding AD from a mechanical perspective will establish a new direction to observe immune mechanisms. This strengthens the hypothesis and provides a novel perspective on how the mechanical load and mechanical stiffness might act as regulators in various autoimmune disorders, which finally are regulated by integrin-dependent adhesome of immune cells.
